# Immunization with CSP and a RIG-I Agonist is Effective in Inducing a Functional and Protective Humoral Response Against *Plasmodium*

**DOI:** 10.3389/fimmu.2022.868305

**Published:** 2022-05-20

**Authors:** Ana Rafaela Teixeira, Begoña Pérez-Cabezas, David M. Costa, Mónica Sá, Sylvain Golba, Hélèna Sefiane-Djemaoune, Joana Ribeiro, Izumi Kaneko, Shiroh Iwanaga, Masao Yuda, Moriya Tsuji, Silvia Beatriz Boscardin, Rogerio Amino, Anabela Cordeiro-da-Silva, Joana Tavares

**Affiliations:** ^1^ Host-Parasite Interactions Group, Instituto de Investigação e Inovação em Saúde, Universidade do Porto, Porto, Portugal; ^2^ Instituto de Biologia Molecular e Celular, Universidade do Porto, Porto, Portugal; ^3^ Departamento de Ciências Biológicas, Faculdade de Farmácia, Universidade do Porto, Porto, Portugal; ^4^ Center for Production and Infection of Anopheles, Institut Pasteur, Paris, France; ^5^ Department of Medical Zoology, Mie University Graduate School of Medicine, Mie, Japan; ^6^ Research Institute for Microbial Diseases, Osaka University, Osaka, Japan; ^7^ Aaron Diamond AIDS Research Center, Department of Medicine, Columbia University Irving Medical Center, New York, NY, United States; ^8^ Institute for Investigation in Immunology (iii)-INCT, São Paulo, Brazil; ^9^ Department of Parasitology, Institute of Biomedical Sciences, University of São Paulo, São Paulo, Brazil; ^10^ Unit of Malaria Infection and Immunity, Institut Pasteur, Paris, France; ^11^ Parasite Disease Group, Instituto de Investigação e Inovação em Saúde, Universidade do Porto, Porto, Portugal

**Keywords:** malaria, *Plasmodium*, sporozoites, subunit vaccines, CSP, adjuvants, RLR agonists, TLR3 agonist

## Abstract

Malaria is a major public health concern, as a highly effective human vaccine remains elusive. The efficacy of a subunit vaccine targeting the most abundant protein of the sporozoite surface, the circumsporozoite protein (CSP) has been hindered by difficulties in generating an effective humoral response in both quantity and quality. Using the rodent *Plasmodium yoelii* model we report here that immunization with CSP adjuvanted with 5’ppp-dsRNA, a RIG-I agonist, confers early and long-lasting sterile protection in mice against stringent sporozoite and mosquito bite challenges. The immunization induced high levels of antibodies, which were functional in targeting and killing the sporozoites and were sustained over time through the accumulation of long-lived plasma cells in the bone marrow. Moreover, 5’ppp-dsRNA-adjuvanted immunization with the CSP of *P. falciparum* was also significantly protective against challenges using a transgenic *Pf*CSP-expressing *P. yoelii* parasite. Conversely, using the TLR3 agonist poly(A:U) as adjuvant resulted in a formulation that despite inducing high antibody levels was unable to generate equally functional antibodies and was, consequently, less protective. In conclusion, we demonstrate that using 5’ppp-dsRNA as an adjuvant to vaccines targeting CSP induces effective anti-*Plasmodium* humoral immunity.

## Introduction

Malaria, a vector-borne disease caused by infection with *Plasmodium* spp, is the deadliest parasitic disease in the world, having caused an estimated 241 million cases and 627,000 deaths in 2020 (1). The great efforts expended for the control of malaria have decreased the incidence and mortality of the disease since 2010 (2) but the reduction in the number of cases has stagnated for the past several years, risking the completion of milestones for the eradication of the disease (1).

Vaccines are effective tools for the prevention, control, and eradication of several infectious diseases (3, 4) and an effective vaccine against *Plasmodium* would be vital to achieving malaria eradication. Nonetheless, despite decades of research, a highly effective vaccine remains a critical priority. The most advanced vaccine, RTS,S/AS01, provides 30-50% efficacy in its target age groups against clinical episodes, but not infection caused by *Plasmodium falciparum*, the most dangerous of the malaria parasites of humans (5). Following encouraging results from pilot implementation and phase IV trials conducted in endemic regions of three African countries (6, 7), the vaccine has been approved by the World Health Organization for widespread use among children living in areas with moderate to high transmission of *P. falciparum* (8).

RTS,S/AS01 directs the immune response to the circumsporozoite protein (CSP) of *P. falciparum*, the protein that densely coats the surface of sporozoites, the motile and invasive form deposited in the skin of the mammalian host through the bite of infected mosquitoes (9, 10). The CSP has a central repeat region, which is immunodominant, an N-terminal containing a proteolytic cleavage site, and a C-terminal containing a known cell-adhesive motif and several T-cell epitopes. RTS,S was designed as a virus-like particle (VLP) comprised of two components: 18 copies of the central repeats and the C-terminal domain of *Pf*CSP fused to the hepatitis B virus surface antigen (HBsAg) with extra HBsAg in a 1:4 ratio and is formulated with the potent liposomal adjuvant system AS01 from GlaxoSmithKline (11, 12).

Another formulation targeting CSP has recently reported high levels of efficacy after a phase 2 clinical trial in children in a high transmission area of Burkina Faso (13). This formulation, similar to RTS,S, incorporates VLPs but with a higher density of CSP epitopes (14) and is formulated with Matrix-M™(R21/MM). When administered before the malaria season it demonstrated ≈77% protection against clinical malaria. However, this trial is ongoing to determine vaccine efficacy during a second malaria season and, importantly, its efficacy is also being assessed in phase 3 trials in areas of varying transmission intensities (13). Other formulations in earlier stages of development, include the full-length CSP, Falciparum Malaria Protein 013 (FMP013) administered together with the Army Liposomal Formulation containing QS-21 (ALFQ), which is currently undergoing phase 1 clinical trials after promising safety and immunogenicity preclinical data (15, 16).

The protection provided by RTS,S/AS01 has been linked to high antibody titers against the NANP amino acid repeats and antibody-mediated functions (17, 18), but it wanes over time, as specific antibody titers decrease (17). Likewise, protection in the R21/MM trial has been correlated with high anti-NANP antibodies and these titers also decrease over time, although, unlike RTS,S/AS01, a booster administration 1-year later was effective in increasing specific antibody titers to the levels observed after primary vaccination (13). The requirement of such high and sustained antibody responses to mediate durable protection represents a major challenge (19, 20) and recent research has been focused on understanding and improving the humoral response to CSP.

Adjuvants play an important role in stimulating and increasing the magnitude of the immune response and, as such, they are important tools for improving the response to a CSP-based recombinant vaccine. Preclinical and clinical trials demonstrate the importance of adjuvants in this context. AS01 and ALFQ, both composed of a mixture of the saponin QS-21 and a Toll-like receptor (TLR) 4 agonist formulated in liposomes, have been shown to be important for the induction of CSP-specific interferon-γ (IFN- γ)-producing polyfunctional CD4 T cells and for a better humoral response, both in terms of magnitude and functionality (15, 16, 21). Likewise, increasing the dose of Matrix M™, which is composed of QS-21 contained in lipidic cage-like structures, co-administered with R21 was associated with higher anti-NANP antibody titers and, consequently, with higher vaccine efficacy (13).

We have recently reported that adjuvanting the full length CSP with the TLR3 agonist polyinosinic-polycytidylic acid, or poly(I:C), conferred sterilizing immunity in a *P. yoelii*-rodent model through the production of cytotoxic antibodies which strip off the protective CSP surface coat of sporozoites leaving them vulnerable to their own pore-forming proteins (22).

Poly(I:C) is a synthetic double-stranded RNA capable of activating TLR3 in endosomes (23) as well as Retinoic-acid-Inducible gene I (RIG-I) and the Melanoma Differentiation-Associated gene 5 (MDA5) receptors in the cytosol (24). Poly(I:C) was found to enhance the antibody response and increase the durability of T_H_1 cell immunity and CD8 T cells by inducing the activation of dendritic cells directly and through the production of type I interferons (IFNs) (25, 26). Poly(I:C) has for many years been used in clinical trials as a vaccine adjuvant (27) and it has previously been tested in preclinical models of vaccines against malaria (22, 28). To gain further insights into the potential of RIG-I/MDA5 and TLR3 ligands to induce protective immune responses against malaria, we tested the adjuvant effects of a RIG-I agonist, 5’-triphosphate double-stranded RNA (5’ppp-dsRNA) (29), and a TLR3 agonist, polyadenylic–polyuridylic acid or poly(A:U) (30). Poly(A:U) is a synthetic double-stranded RNA similar to poly(I:C) but composed of repeating units of adenylic acid paired to uridylic acid. Unlike poly(I:C), poly(A:U) activates only TLR3 (31). 5’ppp-dsRNA, on the other hand is a short synthetic double stranded RNA displaying a tri-phosphate moiety in its 5’ and is an agonist of the cytosolic helicase RIG-I (29). Both poly(A:U) and 5’ppp-dsRNA have been described as presenting similar adjuvant effects as poly(I:C), most importantly, boosting antibody production (31–33).

In this work, we immunized mice with *P. yoelii* CSP (*Py*CSP) alone or adjuvanted with either poly(I:C), poly(A:U) or 5’ppp-dsRNA. We studied the protective efficacy of each immunization regimen and the induced immune response to gain further insights on their potential use for malaria vaccines targeting CSP.

## Materials and Methods

### Mice, Parasites and Mosquitoes

*Plasmodium yoelii yoelii* 17XNL expressing GFP under the control of the *pbef1aa* promoter (34) was obtained from the MR4 repository (ATCC number MRA-817). *Plasmodium yoelii yoelii* 17XNL parasites expressing the CSP from *Plasmodium falciparum*, hereby designated as *Pf*CSP/*Py* (35).

*Anopheles stephensi* (Sda500 strain) young female mosquitoes were reared at the Center for Production and Infection of Anopheles (CEPIA) at Pasteur Institute Paris. To produce *Plasmodium* sporozoites, mosquitoes were allowed to feed on infected NMRI mice. Fed mosquitoes were kept in a humidified incubator at 24 ± 0.5°C and were further allowed to feed on non-infected NMRI mice, one week after the infectious feeding. Maintenance and rearing of mosquitoes were performed as described before (36, 37). Infected mosquitoes used for the natural transmission experiments (15 d after the infectious blood meal) were deprived of sucrose for 6 h before experimentation to enhance the rate of mosquito bites.

For immunization experiments, 6-8-week-old female BALB/c mice purchased from Charles River were housed at the IBMC/i3S animal facility under a 12 h alternating cycle of light/dark and access to food and water *ad libitum*. All animal procedures were approved by the i3S Animal Welfare and Ethics Review Body (ORBEA) and the Portuguese national authority (DGAV) for animal health guidelines, according to the statements on the directive 2010/63/EU of the European Parliament and Council.

### Recombinant *P. yoelii* and *P. falciparum* CSP and Peptides

#### Recombinant Proteins

The bacterial expression plasmids of pET21a containing the codon-optimized synthetic genes lacking the sequences for the signal peptide and GPI-anchor signal of *Plasmodium yoelii* circumsporozoite protein (*Py*CSP; accession number P06914; amino acids 23–346) and *Plasmodium falciparum* circumsporozoite protein (*Pf*CSP; accession number XP_001351122; amino acids 27–384, containing just 23x NANP and 3x NVDP repeats), both coupled to a C-terminal hexahistidine tag were employed for the expression of the respective proteins as described before (22). Briefly, the recombinant proteins were expressed in *Escherichia coli* BL21 (DE3) Star (Invitrogen, Carlsbad, CA, USA), on induction with 1 mM isopropyl-β-D-thiogalactoside (IPTG) (NZYTech, Lisbon, Portugal) for 4h at 37°C (*Py*CSP) and 34°C (*Pf*CSP). Purification was performed using Ni-NTA superflow resin (Qiagen, Hilden, Germany) under native conditions for *Py*CSP and denaturing conditions for *Pf*CSP, according to the manufacturer’s instructions. The purified *Py*CSP was dialysed using a PD-10 column (GE Healthcare, Wauwatosa, Wisconsin) against phosphate buffer saline (PBS) pH 7.4. Buffer exchange of *Pf*CSP to PBS was performed using an Amicon^®^ Ultra-15 Centrifugal Filter Unit (MWCO 30 kDa; Merk-Millipore, Burlington, MA, USA). The protein batches were subjected to Triton X-114 phase separation for endotoxin removal (38). Endotoxin levels in the final protein preparations were quantified using the Pierce LAL Chromogenic Endotoxin Quantitation kit (Thermo Fisher Scientific, Waltham, MA, USA), according to the manufacturer’s instructions. All protein batches used for immunization had endotoxins levels below 1 EU/mL. Protein concentration was determined by semi-quantitative SDS–polyacrylamide gel electrophoresis using bovine serum albumin (BSA) as a standard.

#### Recombinant Peptides

The sequences for *Py*CSP N-terminal (amino acids 23-140) and C-terminal (amino acids 296-346) were amplified by a polymerase chain reaction from the pET21a_*Py*CSP plasmid, using a recombinant *Taq* polymerase (Invitrogen) according to manufacturer’s instructions. The reaction was performed in such a way as to insert *Hind*III and *Xho*I restriction sites at 5’ and 3’, respectively, and add two nucleotides at 5’ for in frame expression, using the following primers, *HindI*II_Nterm: 5’- AAGCTTGCTACGGCCAGCAAAA-3’ and *Xho*I_Nterm: 5’- CTCGAGGTCCACGTTCTCGT-3’; *HindI*II_Cterm: 5’-AAGCTTGCCCCGACGGGAA-3’ and *Xho*I_Cterm: 5’-CTCGAGGCTGCTGCACTT-3’. The fragments were cloned into a pET21d expression vector (Invitrogen), using the *Hind*III and *Xho*I restriction sites. The peptide representing the central repeat region of the protein (amino acids 141-295) was cut from the pET21a_*Py*CSP vector using *Xma*I and *Sac*II and cloned into a pCR-XL-TOPO plasmid (Invitrogen) which had been modified to include some new restriction sites (including *Xma*I, *Sac*II, and *Not*I) in the TOPO^®^ cloning site and from which the Zeocin resistance open reading frame had been excised. After cloning, the fragment was excised from the plasmid using *Hind*III and *Xho*I and cloned onto the pET21d expression plasmid. The conditions used for the expression and purification of the peptides were the same as the ones used for the recombinant full-length *Py*CSP described above.

#### Synthetic Peptides

Synthetic peptides of the repeat region of *Py*CSP were purchased from NZYTech. The peptides used were: 3x the unit sequence of the major repeats, (QGPGA)_3_; 3x the unit sequence for the minor repeats, (PPQQ)_3_; and the first 14 amino acids of the unannotated region immediately following the minor repeats, PDGNNNNNNNNGNN.

### Mice Immunizations

Poly(A:U), poly(I:C) HMW, and 5’ppp-dsRNA (*In vivo*Gen, San Diego, CA, USA) were prepared according to the manufacturer’s instructions. Female BALB/c mice, were immunized with 100 μL of vaccine formulation, administered by intraperitoneal injection. This vaccine formulation included 2 or 5 µg of recombinant protein in saline or combined with either 50 μg of free poly(A:U) or poly(I:C), or 2.5 μg of 5’ppp-dsRNA complexed with GenJet Plus Transfection Reagent (Signagen Laboratories, Rockville, MD, USA). Age-matched female BALB/c mice receiving saline, or the adjuvants alone were used as negative controls. The administration of 5’ppp-dsRNA with transfection reagent was performed according to the latter’s manufacturer’s instructions. Briefly, both RNA and transfection reagent were diluted in a 5% (m/v) glucose solution in water and mixed to a ratio of 2:1 of GenJet (µL) to RNA (µg). The mixture was incubated for 10 minutes at room temperature before adding the required amount of protein diluted in saline or saline alone, to complete the final injection volume. For passive immunization experiments, female BALB/c mice received 100 µL of sera from actively immunized animals with high anti-*Py*CSP titers (≥10^5^), intravenously, 3 days or 24 h before the challenge. Age-matched control female BALB/c mice received the same volume of sera from adjuvant-control animals.

### Sporozoite and Mosquito Challenge

*P. yoelii* 17XNL GFP and *Pf*CSP/*Py* sporozoites were collected from the salivary glands of infected *A. stephensi* mosquitoes 15 days post-infectious feeding. The challenge was performed by the inoculation of 5,000 sporozoites in 1µL of saline in the skin of the footpad using a 35–36 G needle with a NanoFil syringe (World Precision Instruments, Saratosa, FL, USA). The intravenous challenge was performed by the inoculation of 250 or 2,500 sporozoites, in 100 µL, in the tail vein using an Omnican 50^®^ U-100 insulin syringe (B. Braun Medical Inc, Bethlehem, PA, USA).

Mice challenge by mosquito bites was performed using *A. stephensi* mosquitoes infected with *P. yoelii* 17XNL GFP, 15 days post-infectious feeding. Mice anesthetized with a mixture of ketamine (125 mg/kg) and xylazine (12.5 mg/kg) were bitten by 20-30 mosquitoes, for a total of 20 consecutive minutes, rotating positions regularly. After the experiment, the number of fed mosquitoes was calculated by visual inspection of the cages, and the prevalence of infection in mosquitoes evaluated by dissection of the salivary glands.

The course of infection was monitored on Giemsa-stained thin blood films by enumerating the percentage of infected red blood cells with asexual parasites (parasitemia). Animals were followed for infection from days 3 to 12 after the challenge. Sterile protection was defined as the absence of blood stage infection at day 12.

### ELISAs for Determination of Antibody Titers Against Recombinant CSP or CSP Peptides

Mouse sera were collected from immunized animals from peripheral blood at the indicated time points. The blood was allowed to clot and was centrifuged for 10 min at 4000 rpm. The sera were stored at -20°C until use.

For the detection of anti-CSP antibodies, high-binding 96-well flat-bottom plates (Greiner Bio-one, Kremsmünster, Austria) were coated overnight with 1 μg mL^-1^ of recombinant CSP or recombinant peptides in carbonate buffer [0.1 M NaHCO3]. The next day the plates were blocked with 1% (m/v) gelatin from porcine skin in PBS, for 1 h at 37°C and washed with 0.1% (v/v) Tween-20 in PBS. Serial dilutions of sera were incubated in PBS 1% gelatin, for 1h at 37°C. After washing, plates were incubated with a horseradish peroxidase-conjugated goat anti-mouse antibody specific for either IgG (total), IgG2a, IgG2b, or IgG1 (1:5,000, Southern Biotech, Birmingham, AL, USA). Plates were washed and revealed using o-phenylenediamine dihydrochloride (OPD; Sigma, St Louis, Mo, USA), according to the manufacturer’s instructions. The reaction was stopped with 3M HCl and the plates read at 492nm in a Synergy 2 microplate reader (Biotek Instruments, Winooski, Vt, United States) with the Gen5 software (Biotek Instruments). Sera antibody titers were determined by plotting the O.D. values against the respective dilution, and curve fitting the data with a power series curve used to determine the dilution at which O.D. equals 0.1 which we have established as our cut-off point for the presence of specific antibodies (39).

### ELISAs for Determination of IgG Against Synthetic CSP Peptides

For the detection of anti-peptide antibodies, high-binding 96-well flat-bottom plates Nunc MaxiSorp (Thermo Fisher Scientific) were coated overnight with 5 μg mL^-1^ of each peptide in PBS. The next day, plates were blocked with 1% bovine serum albumin (BSA) in PBS, for 1 h at 37°C and washed with 0.02% (v/v) Tween-20 in PBS. Single dilutions of the sera were performed in PBS 0.25% BSA and incubated, in duplicate, for 1h at 37°C followed by incubation with a horseradish peroxidase-conjugated goat anti-mouse IgG (1:5,000, Southern Biotech). Plates were washed and revealed using OPD (Sigma), according to the manufacturer’s instructions. The reaction was stopped with 3M HCl and the plates read at 492nm in a Synergy 2 microplate reader (Biotek Instruments) with the Gen5 software (Biotek Instruments).

### T Cell Depletion

Rat anti-mouse CD4 (IgG2b; clone GK1.5) and anti-mouse CD8 (IgG2b, clone 2.43) monoclonal antibodies were produced and purified from the supernatant of the culturing of the respective hybridomas. These hybridomas were kindly provided by Prof. Rui Apelberg from Universidade do Porto. The hybridomas were expanded in high-glucose Dulbecco’s Modified Eagle’s Medium (DMEM; Lonza, Basel, Switzerland) supplemented with 10% premium fetal bovine serum (FBS; Biowest, Nuaillé, France) and then transferred to serum-free EX CELL hybridoma medium (Sigma), where they were maintained until viability reached ≤10%. The supernatant was recovered and filtered. The monoclonal antibodies were purified by affinity chromatography using HiTrap™ Protein G HP 1 mL columns (GE Healthcare) according to the manufacturer’s instructions. Buffer exchange to PBS was performed using PD-10 columns (GE Healthcare).

For depletion, animals were co-administered with 100 µg of each antibody, three and one days before the challenge. Control animals were administered with 200 µg of rat IgG2b isotype control anti-keyhole limpet hemocyanin (clone LFT-2) monoclonal antibody (BioXCell, Lebanon, NH, USA). Cell depletion in the peripheral blood was confirmed before challenge by analyzing CD3+ (clone 17A2, BioLegend, San Diego, CA, USA), CD3+ CD4+ (clone RM4-5, BioLegend) and CD3+CD8+ (clone 53-6.7, BioLegend) T-cell populations within whole singlet lymphocytes.

### *In Vitro* Assays Using Sporozoites

For *in vitro* sporozoite assays, sera pools of *Py*CSP-immunized animals were used. For cytotoxicity assays, 8,000 to 10,000 sporozoites were collected in base DMEM media, which was after complemented with 10% (v/v) of serum, FBS or FBS plus 100 µg/mL of anti-*Py*CSP repeats monoclonal antibody J6 (22) (for negative and positive conditions, respectively), in the presence of 5 µg/mL of propidium iodide. The suspensions were incubated for 45 minutes at 37°C and then transferred to a µ-Slide 18 Well – Flat (IBIDI, Gräfelfing, Germany), centrifuged at 500 g for 5 minutes, and imaged immediately in an IN CELL Analyzer 2000 using the 40x objective (GE Healthcare).

For CSP reaction assays, 8,000 to 10,000 sporozoites were collected in base DMEM media, which was after complemented with 5% (v/v) of serum, FBS or FBS plus 100 µg/mL of anti-*Py*CSP repeats monoclonal antibody J6. The suspensions were transferred to a µ-Slide 18 Well – Flat (IBIDI), which was centrifuged at 500 g for 5 minutes and then incubated at 37°C, 5% CO_2_ for 45 minutes. After this incubation, the plates were again centrifuged, the supernatant removed and the sporozoites fixated by incubating them for 45 minutes at room temperature with PBS 4% paraformaldehyde. After washing with PBS, the slides were stored at 4°C until stained. For the staining, the wells were blocked with PBS-10% FBS and then incubated in anti-*Py*CSP mouse sera (1:1,000). After washing, the wells were further incubated in anti-mouse Alexa Fluor 488-conjugated goat antibody (1:1,000, BioLegend). The samples were imaged in IN CELL Analyzer 2000 (GE Healthcare), using the 40x objective. The images were analyzed for the percentage of parasites showing signs of CSP reaction and the area of the precipitate was mapped manually in FIJI/ImageJ (40).

### ELISpots

Nunc MaxiSorp 96-well flat-bottom plates (Thermo Fisher Scientific) were coated overnight with 5 μg mL^-1^ of recombinant *Py*CSP in carbonate buffer. The next day the plates were blocked with PBS-1% BSA, for 1 h at room temperature. Serial dilutions of spleen and bone marrow single-cell suspensions were prepared in fully supplemented Roswell Park Memorial Institute (RPMI) 1640 medium (Lonza) and incubated in the coated wells for 6 h at 37°C in 5% CO_2_. Cells were lysed with water and the wells washed [0.05% (v/v) Tween-20 in PBS] and incubated overnight at 4°C in horseradish peroxidase-conjugated goat anti-mouse IgG (1:5,000; Southern Biotech). Plates were washed and visualized with the addition of tetramethylbenzidine (TMB; Mabtech, Stockholm, Sweden) according to the manufacturer’s instruction. Spots were counted manually after imaging the plates in a IN CELL Analyzer 2000 (GE Healthcare) with the 5x objective. The number of antibody secreting cells per million total cells was determined by counting the spots of the lowest dilution containing between 20-30 spots.

### Flow Cytometry

Single-cell suspensions of the spleen and bone marrow were prepared and stained with phenotypic and functional markers to assess the germinal center (GC) reaction, T follicular helper (T_FH_), and humoral response. All antibodies were purchased from BioLegend, unless stated otherwise. Monoclonal antibodies against mouse antigens used include FITC-conjugated anti-B220 (clone RA3-6B2), anti-CD8 (clone 53-6.7), anti-CD95 (clone Jo2; BD Pharmingen), PE-conjugated anti-CD4 (clone H129.19) and CXCR5 (clone L138D7), PERCP/Cy5.5-conjugated anti-CD4 (clone RMA4-4), PE/Cy7 conjugated anti-PD1 (clone RMP1-30), Alexa Fluor 700-conjugated anti-CD44 (clone IM7), APC-conjugated anti-CD138 (clone 281-2) and Brilliant Violet 510-conjugated anti-B220 (clone RA3-6B2). Alexa Fluor 594-conjugated peanut agglutinin (PNA; Invitrogen) was also used. Cells were analyzed in a BD FACSCanto II flow cytometer (BD Biosciences, Franklin Lakes, NJ, U.S.) running BD FACSDiva Software version 6.1.3 (BD Biosciences), and results analyzed using FlowJo version 10.7.1 (FlowJo LLC, Ashland, OR, USA). GC B cells were defined as B cells (B220^+^ CD4^-^) expressing CD95 and PNA. T_FH_ cells were defined as CD4 T cells (B220^-^ CD4^+^ CD44^+^) expressing CXCR5, along with PD-1. These cell populations were assessed in the spleen. Long lived plasma cells (LLPCs) were characterized, in the bone marrow, as B220^-/low^ CD138^+^ cells.

### Cytokine Analysis

Splenocytes from immunized and adjuvant control animals were plated in U-bottom cell culture 96 well plates (Orange Scientific, Braine-l’Alleud, Belgium), and stimulated *ex vivo* with 10 µg/mL of recombinant *Py*CSP for 72 h, at 37°C, 5% CO_2_. Supernatants were collected and stored at -80°C until use. Cytokines were quantified in the supernatants using commercial kits, according to manufacturer’s instructions: Mouse IL-10 DuoSet ELISA (R&D Systems, Minneapolis, Minn, USA), mouse IL-4, IL-6, IL-2, TNF-α and IFN-γ ELISA MAX (BioLegend).

Sera of immunized and adjuvant-control animals was collected 5 hours post prime immunization and analyzed using the LEGENDplex™ Mouse Anti-Virus Response (13-plex) Panel (BioLegend), according to manufacturer’s instructions using a BD FACSCanto II flow cytometer (BD Biosciences) running BD FACSDiva Software version 6.1.3 (BD Biosciences). Results were analyzed using FlowJo version 10.7.1 (FlowJo LLC) and the concentration of the cytokines IFN-γ, TNF-α, IL12(p70), IL-1β, GM-CSF, IL-10, IFN-β, IFN-α, IL6, and chemokines CXCL1 (KC), CCL2 (MCP-1), CCL5 (RANTES) and CXCL10 (IP-10) determined, in pg/mL.

### Immunofluorescence Staining and Microscopy

Spleens were snap-frozen in optimum cutting temperature compound (Killik, Bio-Optica, Milan, Italy), and 8 µm sections were mounted on SuperFrost Plus microscopy slides (Thermo Fisher Scientific) before fixation in 100% methanol for 5 min at room temperature. Slices were blocked with 5% BSA and stained with Alexa Fluor 488 conjugated anti-mouse B220 monoclonal antibody (clone RA3-6B2), Alexa Fluor 594 conjugated peanut agglutinin (PNA) (Invitrogen), and Alexa Fluor 647-conjugated anti-mouse CD4 (clone GK1.5) for 1 hr at room temperature. All antibodies and reagents were purchased from Biolegend, unless stated otherwise. Slides were mounted in Fluorescence Mounting Medium (Dako, Glostrup, Denmark). Images of individual follicles were acquired with a 10x objective on a Zeiss Axio Imager Z1 microscope (Carl Zeiss, Oberkochen, Germany) and images of the full sections were obtained by imaging the slides in an IN CELL Analyzer 2000 (GE Healthcare), with 2x2 binning and 2.5D deconvolution imaging in 10x objective. The area of B220 and PNA staining, as well as the number of naïve and PNA^+^ follicles, were quantified using FIJI/ImageJ (40).

### Statistical Analysis

Statistical significance was determined with Log-Rank (Mantel-Cox) test for the incidence of blood stage infection, unpaired two-tailed *t*-test or 2-way ANOVA with Tukey’s multiple comparisons test for comparison of anti-CSP IgG titers, or 2-way ANOVA with Bonferroni’s multiple comparison test for comparison of functional *in vitro* sporozoite assays, cell populations, and cytokine concentration (comparing between different adjuvanted formulations and between formulations with and without protein for each adjuvant). Analysis was performed using GraphPad Prism version 7 (GraphPad Software, San Diego, CA, USA) or RStudio running R version 3.4.2 for Mac OS X.

## Results

### Immunization of Mice With *Py*CSP and 5’ppp-dsRNA But Not Poly(A:U) Confers Short-Term Sterile Protection Against a Stringent Sporozoite Challenge

**Figure 1:**
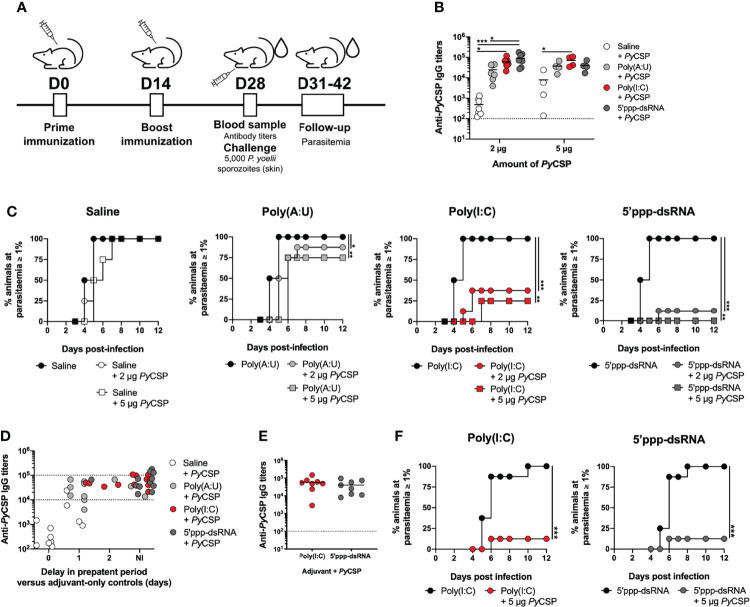
Short-term protection conferred by immunization with *Py*CSP in combination with different adjuvants. **(A)** Immunization and challenge conditions. **(B-D)** Protection to a skin challenge in control mice (n = 8, two independent experiments) and mice immunized with 2 µg (n = 8, two independent experiments) or 5 µg (n = 4, one experiment representative of three, B or one independent experiment, C) of *Py*CSP in saline or in combination with different adjuvants, 2 weeks post booster immunization. **(B)** Total anti-*Py*CSP IgG titers in the sera of immunized animals. Symbols represent individual values and black lines the mean of each group. Dotted line represents the minimal detectable titer. **(C)** Incidence of blood stage infection in control and immunized animals. **(D)** Total anti-*Py*CSP IgG titers *versus* the delay in prepatent period, in days, in immunized mice. The interval for which sterile protection is independent of antibody titers is represented between dotted lines. **(E, F)** Protection to mosquito bite challenge in control mice (n = 8) and mice immunized with *Py*CSP in saline or in combination with poly(I:C) or 5’ppp-dsRNA (n = 8, two independent experiments for each group), 2 weeks post booster immunization. **(E)** Total anti-*Py*CSP IgG titers in the sera of immunized animals. Symbols represent individual values and black lines the mean of each group. Dotted line represents the minimal detectable titers. **(F)** Incidence of blood stage infection in immunized and control animals. Statistical significance was determined using two-way ANOVA with Tukey’s multiple comparisons test (B) or using a two-tailed unpaired *t*-test (E). Survival curves were plotted using Kaplan-Meier plot and statistical significance was determined using the Log-Rank (Mantel-Cox) test (C and F). *p ≤ 0.05; **p ≤ 0.01; ***p ≤ 0.001.

BALB/c mice were immunized with 2 µg or 5 µg of recombinant *Py*CSP in saline or combined with one of the following adjuvants: poly(A:U), poly(I:C) or 5’ppp-dsRNA. Animals administered just saline or adjuvant were used as controls. All animals were boosted two weeks later and short-term protection was tested upon inoculation with *P. yoelii* sporozoites two weeks after booster immunization. Mice were followed for blood stage infection from day 3 post-challenge onwards until parasitemia reached 1% or, in the absence of blood stage infection, until 12 days after the challenge (**Figure 1A**).

The immunization protocols using 2 µg of *Py*CSP in combination with poly(I:C) or 5’ppp-dsRNA induced significantly higher specific IgG antibody titers compared with animals immunized with protein alone, as measured immediately before the challenge (**Figure 1B**). Interestingly, animals immunized with *Py*CSP in combination with 5’ppp-dsRNA showed also significantly higher titers of specific antibodies than those immunized with *Py*CSP adjuvanted with poly(A:U). Immunization with 5 µg of *Py*CSP increased specific IgG titers when using the protein alone but not when using adjuvants, such that only the immunization in combination with poly(I:C) generated significantly higher titers of specific antibodies when compared to the protein alone (**Figure 1B**). Similar results were observed when analyzing the titers of the specific IgG isotypes IgG1, IgG2a, and IgG2b for 2 µg (**Supplementary Figure 1A**) and 5 µg (**Supplementary Figure 1B**) of *Py*CSP, although significance between adjuvanted and non-adjuvanted immunizations was only achieved for specific IgG1 antibody titers.

Efficacy was assessed by the microinjection of 5,000 *P. yoelii* sporozoites in the skin. All mice immunized with the protein in saline became infected, with no significant delay on the day in which parasitemia ≥ 1% was reached, reflecting no delay in the prepatent period when compared to the respective controls (**Figure 1C**, Saline). Mice immunized with the protein plus poly(A:U) registered 12.5% and 25% of sterile protection for the protocols using 2 µg and 5 µg of *Py*CSP, respectively. However, a significant delay in the prepatent period, of 1-2 days, is seen in infected immunized mice when compared to their respective adjuvant controls, reflecting a degree of protection that, while not enough for sterility, represents a ~10-100-fold decrease in liver parasite burden (41) [**Figure 1C**, Poly(A:U)]. Higher protection is observed in animals vaccinated with *Py*CSP and poly(I:C), which registered 62.5% and 75% rates of sterile protection when using 2 µg or 5 µg of recombinant protein, respectively, as well as a delay, of 1-2 days, in the prepatent period for immunized mice that became infected [**Figure 1C**, Poly(I:C)]. Yet, the highest percentages of sterile protection were achieved when mice were immunized with 2 µg or 5 µg *Py*CSP and 5’ppp-dsRNA, 87.5% and 100%, respectively. The small percentage of animals that became infected registered, nonetheless, a delay of 2 days in the prepatent period (**Figure 1C**, 5’ppp-dsRNA).

Considering the differences in protective efficacy between the formulations and regarding the average differences seen in terms of anti-*Py*CSP antibody titers for poly(A:U) and 5’ppp-dsRNA (**Figure 1B**), we plotted the specific IgG titers of each mouse against their protective status, of either non-infected (NI) or represented by the delay, in days, in the prepatent period *versus* their respective adjuvant controls. This representation shows that elevated titers (>10^5^) are necessary to ensure sterile protection. However, for animals below this threshold but within the range of 10^4^-10^5^ specific IgG titers, those that were immunized with *Py*CSP and 5’ppp-dsRNA are far more likely to be non-infected after challenged, when compared to animals immunized with the poly(A:U) adjuvant. Of the 9 animals immunized with *Py*CSP and 5’ppp-dsRNA in such a situation, 8 were sterilely protected (≈89%), 6 of 10 for poly(I:C) (60%) *versus* just 2 non-infected animals of 9 immunized with *Py*CSP and the poly(A:U) adjuvant (≈22%) (**Figure 1D**).

As immunizations combining the recombinant *Py*CSP with either of the RLRs agonists, poly(I:C) or 5’ppp-dsRNA, showed significant rates of sterile protection against the challenge, *via* microinjection in the skin, of 5,000 *P. yoelii* sporozoites, we tested the efficacy of these protocols against a mosquito bite challenge, performed 2 weeks after booster immunization. Mice immunized with 5 µg of *Py*CSP in combination with either poly(I:C) or 5’ppp-dsRNA, following the standard protocol (**Figure 1A**), presented as expected, high specific IgG titers in their sera (**Figure 1E**). All control animals that received only the adjuvants became infected under these conditions, while the rate of sterile protection for immunized animals from both protocols was 87.5%, with infected mice displaying a 1-day delay in the prepatent period (**Figure 1F**).

### The Skin Is the Main Site of Protection After Immunization With *Py*CSP in Combination With 5’ppp-dsRNA and Antibodies Play a Central Role in This Process

Protection conferred by immunization with *Py*CSP in combination with poly(I:C) was previously shown to target the extravascular sporozoites deposited in the skin, there being the critical site for the sterile protection mediated by cytotoxic anti-CSP antibodies (22). To further characterize 5’ppp-dsRNA as an adjuvant for malaria vaccines, we tested whether the protection conferred by this immunization strategy was also affected by the route of sporozoites’ inoculation. Mice were actively immunized and boosted, as previously described, with 5 µg of *Py*CSP plus 5’ppp-dsRNA. Immunized animals were challenged, 2 weeks post booster immunization, by intravenous injection of 2,500 or 250 *P. yoelii* sporozoites. Intravenous inoculation of 2,500 sporozoites results in the same liver parasite load as the infection of 5,000 sporozoites delivered in the skin and can be, therefore, considered a stringent challenge (22).

**Figure 2:**
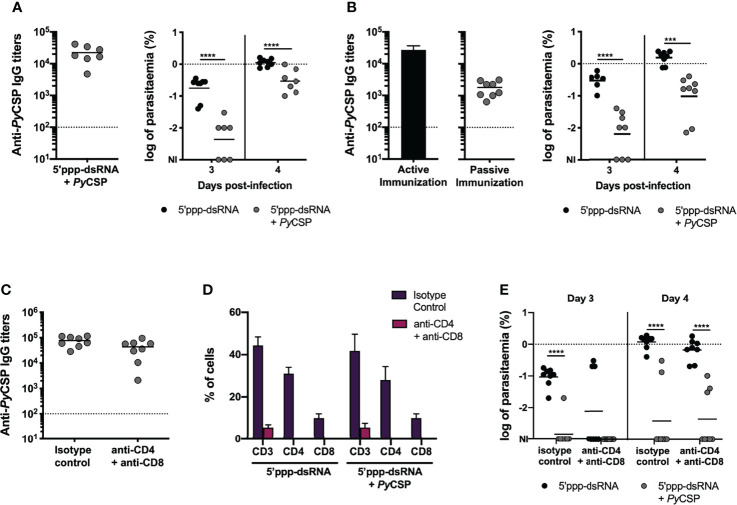
Characterization of the mechanisms involved in short-term protection induced by immunization with *Py*CSP and 5’ppp-dsRNA. **(A)** Protection to intravenous challenge in control mice (n = 8) and mice immunized with *Py*CSP in combination with 5’ppp-dsRNA (n = 7, two independent experiments), 2 weeks post booster immunization. *Left:* Anti-*Py*CSP antibody titers in the sera of immunized mice. Symbols represent individual values and black lines the mean of each group. Dotted line represents the minimal detectable titers. *Right:* Log of parasitemia in immunized and control mice on days 3 and 4 post challenge with 2,500 sporozoites. Symbols represent individual values and black lines the mean of each group. The dotted line marks parasitemia of 1%. **(B)** Protection in mice which received the sera of control animals (n = 8), or sera of mice immunized with *Py*CSP plus 5’ppp-dsRNA (n = 8, two independent experiments). *Left:* Anti-*Py*CSP antibody titers in the pool of sera transferred to naïve mice (bar, “Active Immunization”) and in the sera of immunized mice 3 days after transfer (symbols, “Passive Immunization”). The bar represents the mean ± SD obtained from two independent determinations. Symbols represent individual values and the black line the mean for the group. Dotted line represents the minimal detectable titers. *Right:* Log of parasitemia in control and immunized mice, at days 3 and 4 after skin challenge with 5,000 *P.yoelii* sporozoites. Symbols represent individual values and black lines the mean of each group. The dotted line marks parasitemia of 1%. **(C–E)** Protection to skin challenge in control and 5’ppp-dsRNA-adjuvanted *Py*CSP-immunized mice which received isotype control antibody (n = 8, two independent experiments for both groups) or were depleted of CD4 and CD8 T cells (n = 8, two independent experiments for both groups), 2 weeks post booster immunization. **(C)** Anti-*Py*CSP IgG titers in the sera of immunized animals. Symbols represent individual values and black lines the mean of each group. Dotted line represents the minimal detectable titers. **(D)** Percentage of total CD3+, CD3+CD4+ and CD3+CD8+ T cells, within single cell lymphocyte population, in the peripheral blood of immunized and control animals. Bars represent the mean ± SD of the percentage for each group and cell population. **(E)** Log of parasitemia in control and immunized mice, on days 3 and 4 post infection. Symbols represent individual values and black lines the mean of each group. The dotted line marks parasitemia of 1%. Statistical significance was determined using 2-way ANOVA with Tukey’s multiple comparisons test (A, B, D and E) or two-tailed unpaired *t* test (C). NI, non-infected. ***p ≤ 0.001; ****p ≤ 0.0001.

Despite the high circulating specific IgG titers at the time of the challenge (**Figure 2A**, left), no sterile protection was achieved using 2,500 sporozoites, since parasitemia was detected in all animals by day 4 (**Figure 2A**, right), as opposed to 100% sterility achieved for the same protocol when performing an equivalent skin challenge (**Figure 1C**, 5’ppp-dsRNA). However, a delay in the prepatent period was detected, with significantly lower parasitemia values on days 3 and 4 post-challenge for immunized animals when compared to controls (**Figure 2A**, right). The less stringent challenge with 250 sporozoites was enough to infect all control animals. However, immunized animals registered a 25% rate of sterile protection (**Supplementary Figure 2A**, right). Again, animals that became infected showed a significantly decreased parasitemia in immunized mice *versus* controls on days 5 and 6 post-challenge (**Supplementary Figure 2A**, right).

The almost complete abrogation of sterile protection in response to an intravenous challenge, regardless of parasite inoculum, reveals that protective mechanisms generated in response to immunization with *Py*CSP in combination with 5’ppp-dsRNA are most likely targeting the sporozoites while in the skin, as opposed to when they are on their way to or on the liver itself.

To study the role of antibodies in the protection, a pool of sera from mice actively immunized with *Py*CSP in combination with 5’ppp-dsRNA was transferred into naïve mice and three days after the transfer, animals were challenged by the microinjection of 5,000 *P. yoelii* sporozoites in the skin. Before the challenge, circulating specific IgG titers were determined in the passively immunized animals and those were observed to be lower than the titers found 2 weeks after active booster immunization (**Figure 2B**, left). All animals became infected following the challenge, but passively immunized animals displayed significantly lower parasitemia at days 3 and 4 post-challenge when compared to control mice (**Figure 2B**, right). To test whether the lower degree of protection achieved by passive immunization could be related to the lower specific IgG titers present in the circulation of these mice, animals were challenged with the inoculation of sporozoites in the skin, just 24 hours after the sera transfer, when the titers of circulating antibodies were similar to those seen in active immunization (**Supplementary Figure 2B**, left). However, the rate of protection observed in these animals was similar to the one determined in the previous passive immunization experiments, in that no sterility was achieved but protection was observed in terms of a significantly lower blood stage parasitemia in the immunized mice when compared to their controls in days 3 and 4 post-challenge (**Supplementary Figure 2B**, right).

We next evaluated the involvement of effector T cells in the protective efficacy of this immunization strategy. Mice were immunized and boosted, as described before, with 5 µg of *Py*CSP in combination with 5’ppp-dsRNA and challenged two weeks post booster immunization (**Figure 1A**). Mice administered just the adjuvant were used as controls. Three and one days before challenge, animals were administered a mixture of anti-CD4 (clone GK 1.5) and anti-CD8 (clone 2.43) monoclonal antibodies for the depletion of T cells or an isotype control antibody. The challenge was performed by the microinjection of 5,000 *P. yoelii* sporozoites in the skin.

The sera titers of specific IgG were high, as expected, and not different between depleted and non-depleted groups at the time of challenge (**Figure 2C**). Successful depletion of CD4+ and CD8+ T cells was evaluated by flow cytometry analysis of the CD3+ cell compartment in peripheral blood, which confirmed that animals administered with the combination of depletion antibodies showed ≥ 99% decrease in the percentage of both CD4+ and CD8+ cells when compared to mice that received the equivalent amount of isotype control antibody (**Figure 2D**).

The efficacy of the immunization protocol was similar between depleted and non-depleted immunized mice, but significantly higher in both when compared to their respective control mice, as measured both by the rate of sterile protection in immunized mice and the parasitemia values, counted on days 3 and 4 post-challenge (**Figure 2E**).

As no differences were observed in the protective efficacy of immunized animals between depleted and intact mice, we conclude that neither CD4+ nor CD8+ T cells play a major effector role in the protection of mice immunized with *Py*CSP in combination with 5’ppp-dsRNA. Protection is likely dependent on antibodies.

### Immunization of Mice with *Py*CSP and the RLRs Agonists Poly(I:C) and 5’ppp-dsRNA Induces Effective Anti-CSP Antibodies

**Figure 3:**
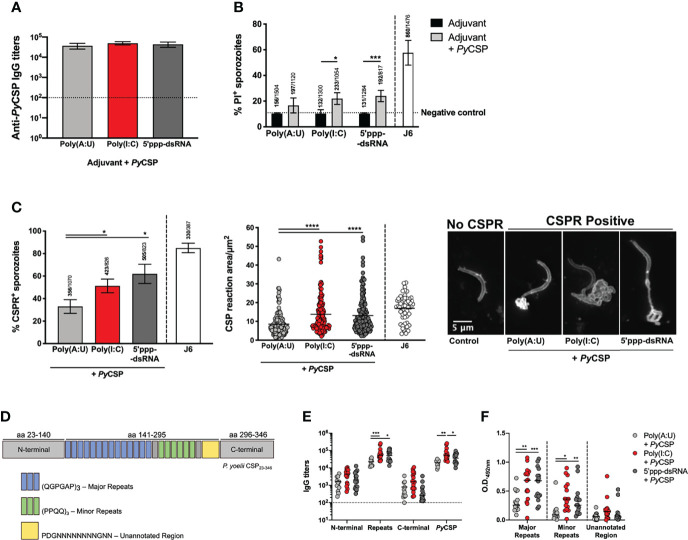
Functionality of the humoral response induced by the immunization of mice with *Py*CSP in combination with different adjuvants. **(A)** Total anti-*Py*CSP IgG titers in sera pools used for the functional assays. Bars represent the mean titers ± SD of two independent determinations. Dotted line represents the minimal detectable titers. **(B)** Cytotoxicity assays. Sporozoites were incubated with each sera pool or the anti-*Py*CSP repeats monoclonal antibody J6 [a cytotoxic monoclonal antibody previously characterized (22)], in the presence of propidium iodide (PI). Bars represent the mean ± SD of the percentage of PI+ sporozoites counted from three independent assays; above the bars are indicated the number of PI+ sporozoites/the total number of sporozoites counted. The dotted line represents the basal percentage of PI+ sporozoites. **(C)** CSP reaction (CSPR) assays. Sporozoites were incubated with each sera pool or the anti-*Py*CSP repeats monoclonal antibody J6 and stained for *Py*CSP. *Left*: Percentage of CSPR+ sporozoites represented as mean ± SD from three independent assays; indicated above the bars are the number of CSPR+ sporozoites/total number of sporozoites counted. *Center*: area of CSPR in µm^2^. Symbols represent individual values and black lines the mean of each group, from three independent experiments. *Right:* Representative images of CSPR- and different CSPR+ sporozoites. **(D–F)** Individual sera of mice immunized with *Py*CSP in combination with different adjuvants obtained 2 weeks post booster immunization were tested for their binding to peptides representing different areas of *Py*CSP. **(D)** Schematic representation of recombinant *Py*CSP and the different peptides used in the assays. **(E)** IgG titers against the recombinant *Py*CSP and its peptides. Symbols represent individual values and black lines the mean of each group. Dotted line represents the minimal detectable titers. **(F)** O.D. values at 492nm of the binding of the sera to the major and minor repeats peptides (sera dilution: 1:1000) and the unannotated region (sera dilution: 1:100) of *Py*CSP. Symbols represent individual values and black lines the mean of each group. Statistical significance was determined using one-way ANOVA with Tukey’s multiple comparisons test (A, C) or two-way ANOVA with either Bonferroni’s (B) or Tukey’s (E, F) multiple comparisons test. *p ≤ 0.05, **p ≤ 0.01; ***p ≤ 0.001; ****p ≤ 0.0001.

We have demonstrated that antibodies are central for protection conferred by the immunization with *Py*CSP in combination with 5’ppp-dsRNA. Similarly, such was found to be the case for the protocol using poly(I:C) as adjuvant and, in that case, protection was linked to cytotoxic antibodies which kill sporozoites by inducing extensive precipitation of CSP in a phenomenon known as CSP reaction (CSPR) (22). In our experiment, we have found that the titers of specific antibodies do not, by themselves, fully explain the differences observed in the efficacy of the different immunization strategies (**Figure 1D**). To understand if the quality of the humoral response was playing a role following immunization with *Py*CSP adjuvanted with 5’ppp-dsRNA *versus* poly(A:U), we made pools of sera collected 2 weeks after booster immunization from mice immunized with the recombinant protein in combination with the different adjuvants. Each sera pool was normalized to a similar anti-*Py*CSP IgG titer (**Figure 3A**).

Cytotoxicity of each sera pool was tested, as previously described (22), by incubating *P. yoelii* sporozoites with the sera pools in the presence of propidium iodide (PI). The results show that the percentage of PI positive sporozoites was on average higher when incubation was performed with the sera of animals immunized with *Py*CSP and either poly(I:C) or 5’ppp-dsRNA comparing with sporozoites incubated with sera of animals from *Py*CSP plus poly(A:U) immunized animals but neither difference was statistically significant (**Figure 3B**). Still, incubation with the sera of animals immunized with the protein and either of the RLRs agonists led to statistically significant higher percentages of PI positive sporozoites when compared with incubation in their respective adjuvant-control sera, which is not observed in the conditions using sera from poly(A:U) administered mice (**Figure 3B**).

The capacity of sera pools to induce CSPR was also tested by incubating the sporozoites with the same sera pools and staining them for detection of *Py*CSP. The percentage of sporozoites showing signs of CSPR was significantly higher when incubated with the sera of animals immunized using *Py*CSP plus poly(I:C) or 5’ppp-dsRNA when compared to the poly(A:U) adjuvanted condition (**Figure 3C**, left). Similarly, this reaction was significantly more extensive, as measured by its area, in the sporozoites incubated in the sera of animals immunized with *Py*CSP with poly(I:C) or 5’ppp-dsRNA when compared with sporozoites incubated in the sera of animals immunized with poly(A:U)-adjuvanted formulations (**Figure 3C**, center and right). On the other hand, there were no statistically significant differences when comparing the conditions using the sera of mice immunized with the protein and either of the RLRs agonists (**Figure 3C**).

Neutralizing antibodies generated against CSP were described to principally target the central repeats region of the protein (22, 42, 43). Considering this, we tested whether the different immunization protocols used in this work resulted in antibodies targeting different regions of the protein. Recombinant peptides covering the three major regions of the protein, the N-terminal (aa 23-140), central repeats (aa 141-295), and C-terminal (aa 296-346), were expressed in *E. coli* (**Figure 3D**). Specific IgG titers for each of these regions were determined in the individual sera from mice collected 2 weeks after booster immunization with the different protocols. The sera from mice immunized with *Py*CSP adjuvanted with poly(I:C) show significantly higher titers against the full-length protein compared to animals immunized and adjuvanted with poly(A:U) or 5’ppp-dsRNA, and against the central repeats region when compared only with animals that received *Py*CSP plus poly(A:U). Interestingly, the titers against the central repeats region were also significantly higher in the group of mice adjuvanted with 5’ppp-dsRNA than in the mice adjuvanted with the TLR3 agonist. No significant differences were observed among the different mice groups regarding the recognition of the N-terminal and the C-terminal parts of the protein (**Figure 3E**).

The *Py*CSP central repeat region is composed of two different basic units comprising major and minor repeats (**Figure 3D**). We tested the binding of the antibodies in the sera to synthetic peptides comprising 3 repeats of the base units of the major and minor repeats. As an internal control, a peptide of similar length covering an unannotated region of the protein located between the central repeats and the protein’s C-terminal was used (**Figure 3D**). Results obtained from this assay showed that the sera of mice immunized with the protein in combination with any of the RLRs agonists bind more extensively to both major and minor repeats than the sera obtained from the immunization protocol using poly(A:U) (**Figure 3F**).

Antibodies produced in response to the protective immunization strategy, namely the *Py*CSP adjuvanted with poly(I:C) or, especially, 5’ppp-dsRNA, demonstrate increased functionality over the response to immunization when *Py*CSP is combined with poly(A:U). Antibodies generated following immunization with *Py*CSP combined with either with poly(I:C) or 5’ppp-dsRNA show increased cytotoxicity, probably resulting from their enhanced capacity to induce CSPR and their increased recognition of the central repeats region. Thus, we have linked sterile protection in these immunizations not only to robust antibody production but also to the quality of the response.

### Immunization of Mice With *Py*CSP and the RLRs Agonists Poly(I:C) and 5’ppp-dsRNA Leads to the Accumulation of Long-Lived Plasma Cells in the Bone Marrow

Protection against *Plasmodium*, and particularly against the sporozoite stage, requires sustained antibody production over time. This can be achieved through the generation of long-lived plasma cells (LLPCs), antibody secreting cells (ASCs) formed through somatic hypermutation in the germinal center (GC) reaction, that migrate to the bone marrow, where they continuously secrete antibodies to the bloodstream (44, 45).

To ascertain if our immunization strategies were inducing LLPC accumulation in the bone marrow, mice were immunized as previously described and their spleens and bone marrow were collected to quantify the number of ASCs in both organs, 2- and 12-weeks post booster immunization by B cell ELISpot.

**Figure 4:**
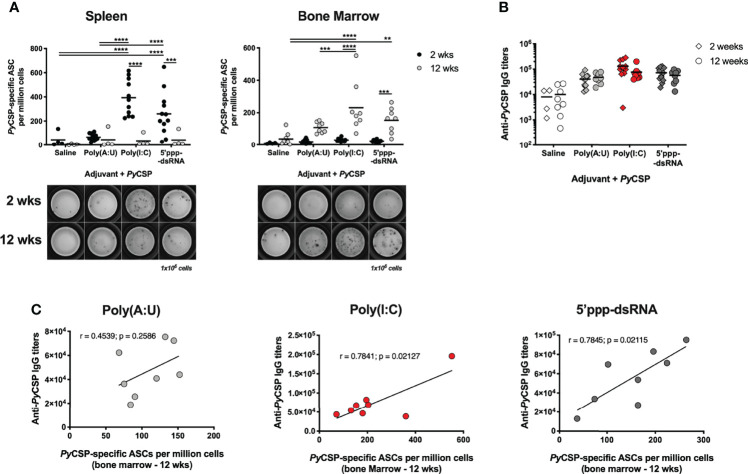
Antibody secreting cells induced by immunization with *Py*CSP in combination with different adjuvants. **(A)** *Py*CSP-specific antibody secreting cells (ASCs) in the spleen (left) and bone marrow (right) of animals immunized with *Py*CSP in saline or in combination with different adjuvants, 2 and 12 weeks after booster immunization. Symbols represent individual values and black lines the mean of each group, obtained from three (2 weeks timepoint, n = 12) and two (12 weeks timepoint, n = 8) independent experiments. Below the graphs, a representative image of the ELISpot for each condition is shown. **(B)** Total anti-*Py*CSP IgG titers of mice immunized with *Py*CSP in saline or in combination with different adjuvants. Symbols represent individual values and black lines the mean of each group. **(C)** Correlation between the total anti-*Py*CSP IgG titers and the number of *Py*CSP specific ASCs in the bone marrow 12 weeks post booster immunization for animals immunized with *Py*CSP and poly(A:U), poly(I:C) or 5’ppp-dsRNA. Person’s correlation coefficients, r, and two-tailed p values were computed for each pair. Statistical significance was determined using two-way ANOVA with either Bonferroni’s (A) or Tukey’s (B) multiple comparisons test. **p ≤ 0.01; ***p ≤ 0.001; ****p ≤ 0.0001.

Mice immunized with *Py*CSP in combination with either poly(I:C) or 5’ppp-dsRNA significantly differentiated more ASCs in the spleen 2 weeks post booster immunization than mice immunized with the protein alone or in combination with poly(A:U). The numbers of these cells in the spleen significantly decreased 12 weeks after booster immunization (**Figure 4A**, left), while the number of ASCs in the bone marrow increased with time (**Figure 4A**, right). Interestingly, the number of LLPCs was significantly higher in animals immunized in the protocols using poly(I:C) or 5’ppp-dsRNA when compared with the protocols using the protein in saline or in combination with poly(A:U) (**Figure 4A**, right). No differences were observed for mice immunized with the *Py*CSP and either of the RLRs agonists.

Considering these results, the presence of GC B cells and T follicular helper (T_FH_) cells in the spleen, and of LLPCs in the bone marrow of these mice were analyzed by flow cytometry, at the same time points. We observed no differences in either the percentage or the number of these cells between the mice immunized in the different protocols including an adjuvant, except for the number of T_FH_ cells which was higher in mice immunized with the protein in combination with poly(I:C) (**Supplementary Figures 3–5**, respectively). Moreover, *ex vivo* cytokine production of splenocytes upon restimulation with the recombinant protein was also studied. Cells from mice immunized with *Py*CSP and 5’ppp-dsRNA produce significantly more IL-4 and IL-10 than cells from mice immunized with any of the other protocols, suggesting a skewed T helper cell type 2 response. (**Supplementary Figure 6A**). Finally, for a more direct visualization of the GC formation and architecture, cryo-sections of spleens obtained 9 days post-immunization were stained for markers of B and T cells (B220 and CD4, respectively) and of GC B cells (PNA) (**Supplementary Figure 6B**). However, no differences were observed in terms of the percentage of GCs and the area and number of B cell follicles and GCs, corrected for the area of the section (**Supplementary Figure 6C**).

Interestingly, differences observed in the number of ASCs between mice immunized with the protein and poly(A:U) and those immunized in combination with the RIG-I adjuvants did not translate into significantly different anti-*Py*CSP IgG titers, at either time point. Importantly, for all protocols, these titers are maintained as late as 12 weeks after booster immunization (**Figure 4B**). The numbers of ASCs per million cells in the bone marrow 12 weeks post booster immunization were plotted against each animal’s specific IgG titers at the same time point. Interestingly, both measures strongly correlated in the case of mice immunized in combination with poly(I:C) or 5’ppp-dsRNA (r = 0.7841, p = 0.0213 and r = 0.7845, p = 0.0211, respectively) but not for the mice similarly immunized but using poly(A:U) as adjuvant (r = 0.4539, p = 0.2586) (**Figure 4C**). It should be noted that, while statistically significant, the correlation observed in poly(I:C)-adjuvanted immunized animals appears to be driven by a single high leverage data point. On the other hand, the correlation for 5’ppp-dsRNA-adjuvanted immunized mice is far more robust.

In brief, we show that the immunization protocols in which *Py*CSP is combined with either poly(I:C) or 5’ppp-dsRNA are successful in the generation and accumulation of LLPCs. Our results strongly suggest that the long-term specific IgG titers produced in response to these protocols, particularly those adjuvanted with 5’ppp-dsRNA, are sustained by these LLPCs.

### Immunization of Mice With *Py*CSP in Combination With 5’ppp-dsRNA Confers Long-Term Sterile Protection Against a Stringent Sporozoite Challenge and Against Reinfection

**Figure 5:**
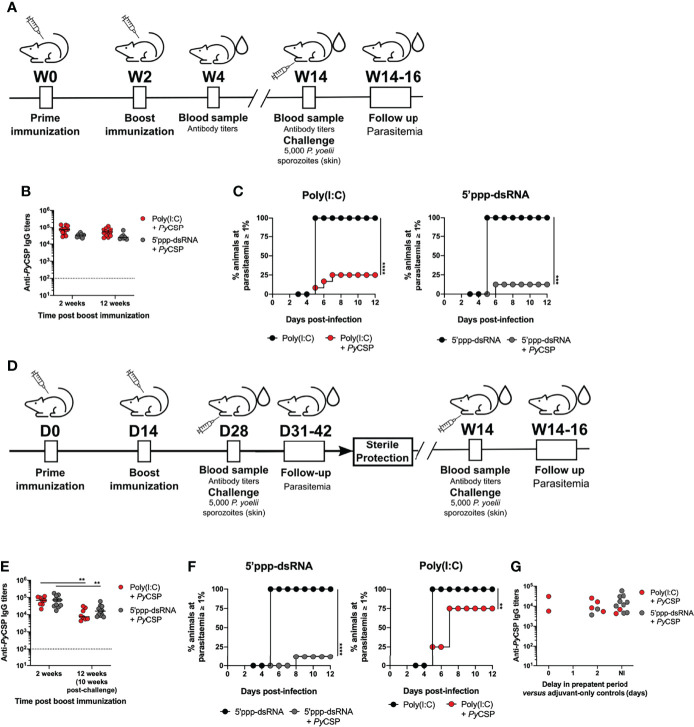
Long-term protection conferred by immunization with *Py*CSP in combination with poly(I:C) and 5’ppp-dsRNA. **(A)** Immunization strategy and challenge conditions. **(B–D)** Long-term protection to a skin challenge in control mice (n = 8) and mice immunized with *Py*CSP in combination with poly(I:C) (n = 8, two independent experiments) or 5’ppp-dsRNA (n=8, two independent experiments), 12 weeks post booster immunization. **(B)** Total anti*-Py*CSP IgG titers 2 and 12 weeks post booster immunization in immunized mice. Symbols represent individual values and black lines the mean for each group. Dotted line represents the minimal detectable titers. **(C)** Incidence of blood stage infection in control and immunized animals. **(D)** Immunized mice which survived a first sporozoite challenge 2 weeks after booster immunization were challenged a second time 10 weeks later. **(E, F)** Protection against a second skin challenge in mice immunized with *Py*CSP in combination with poly(I:C) (n = 7, two independent experiments) or 5’ppp-dsRNA (n = 11, two independent experiments). **(E)** Total anti*-Py*CSP total IgG titers 2 and 12 weeks post booster immunization (10 weeks after first challenge) in immunized mice. Symbols represent individual values and black lines the mean for each group. Dotted line represents the minimal detectable titer. **(F)** Incidence of blood stage infection in control and immunized animals. **(G)** Total anti-*Py*CSP IgG titers *versus* the delay in prepatent period, in days, in immunized re-challenged mice. Statistical significance was determined using two-way ANOVA with Tukey’s multiple comparisons test (B and E). Survival curves were plotted using Kaplan-Meier plot and statistical significance was determined using the Log-Rank (Mantel-Cox) test (C and F). **p ≤ 0.01; ***p ≤ 0.001; ****p ≤ 0.0001.

Animals were immunized with 5 µg of *Py*CSP adjuvanted with either poly(I:C) or 5’ppp-dsRNA, as previously described and long-term protection was tested 12 weeks after booster immunization with the microinjection of 5,000 *P. yoelii* sporozoites in the skin. Mice were followed for parasitemia as described before (**Figure 5A**).

The presence of high anti-*Py*CSP circulating IgG titers induced by the immunization protocols was confirmed. These were not different from the titers measured for the same mice 2 weeks after booster immunization and not significantly different between the adjuvanted immunization protocols at the 12-week time point post booster immunization (**Figure 5B**). Similar results were observed when analyzing the titers of the IgG isotypes IgG1, IgG2a, and IgG2b (**Supplementary Figure 7**). In accordance with the maintenance of high specific IgG titers, we found that immunization with *Py*CSP adjuvanted with either poly(I:C) or 5’ppp-dsRNA was equally protective after 12 weeks from booster immunization. For poly(I:C), the percentage of sterilely protected animals at this later time point was 75% [**Figure 5C**, Poly(I:C)] which is comparable with the 62.5-75% sterile protection rates registered for the short-term challenge [**Figure 1C**, Poly(I:C)]. For the immunization adjuvanted with 5’ppp-dsRNA we observed an 87.5% rate of sterile protection (**Figure 5C**, 5’ppp-dsRNA). Thus, the immunization strategies combining *Py*CSP with the RLR agonists conferred not only early but also long-lasting sterile protection.

In addition to long-term protection, a successful malaria vaccine should also maintain the protection level in the face of multiple challenges, especially in endemic areas. To test the robustness of the protective immunization protocols, we tested their efficacy against a second stringent sporozoite skin challenge.

Mice previously immunized with 2-5 µg of *Py*CSP adjuvanted either with poly(I:C) or 5’ppp-dsRNA and sterilely protected against a first stringent challenge performed 2 weeks after booster immunization (**Figure 1C**, Poly(I:C) and 5’ppp-dsRNA), were again challenged with 5,000 *P. yoelii* sporozoites administered in the skin, 12 weeks after booster immunization. The animals were monitored for signs of blood infection for 12 days after the second challenge (**Figure 5D**).

Immediately before the second challenge, the anti-CSP IgG titers in the sera of the mice immunized with the two different formulations were not different. However, they were, at this time point, significantly lower than the titers in the same animals measured 2 weeks post booster immunization (**Figure 5E**). In contrast, immunized animals which at 12 weeks after booster immunization had not yet been challenged had similar titers to those measured 2 weeks post booster immunization (**Figure 5B**). It is thus apparent that infection with sporozoites leads to a decrease in anti-CSP IgG titers.

Despite the similarity in the circulating specific titers between the two groups of mice, differences in terms of protection to a second challenge were found. Of all the mice immunized with the poly(I:C) adjuvanted formulations and all of which had been sterilely protected against a first stringent challenge, only 25% continued to be sterilely protected to a second challenge (**Figure 5F**, Poly(I:C)). This reduced rate contrasts the rates measured against a first challenge, whether performed 2 or 12 weeks post booster immunization, of 62.5-75% [**Figure 1C**, Poly(I:C)] and 75% [**Figure 5C**, Poly(I:C)], respectively. Nonetheless, infected animals displayed a degree of protection reflected in their increased prepatent period when compared to age-matched control mice that received the adjuvant alone. On the other hand, the rate of sterile protection to re-infection in *Py*CSP plus 5’ppp-dsRNA immunized mice was 82% (**Figure 5F**, 5’ppp-dsRNA). This percentage is in line with that registered for both short-term (**Figure 1C**, 5’ppp-dsRNA) and long-term (**Figure 5C**, 5’ppp-dsRNA) challenges. There was no obvious relation between the differences in protection and the anti-*Py*CSP IgG titers at the time of the second challenge (**Figure 5G**).

Hence, while both protocols in which mice are immunized with *Py*CSP and either of the RLR agonists are equally protective against a long term first challenge, only the use of 5’ppp-dsRNA as adjuvant conferred important sterile protection against a second challenge.

### Immunization With *P. falciparum* CSP in Combination With 5’ppp-dsRNA Confers Protection to Mice Against a Sporozoite Challenge

As a very preliminary assessment of the potential of these formulations for human applications, we immunized BALB/c mice using the *P. falciparum* CSP. Considering the inability of this parasite to infect rodents, we took advantage of a *P. yoelii* parasite, in which endogenous CSP has been replaced by the full-length *Pf*CSP (*Pf*CSP/*Py*) (35).

Recombinant, nearly full length, *Pf*CSP, was expressed and purified (**Supplementary Figures 8A, B**). Mice were immunized with 5 µg of *Pf*CSP in saline or in combination with either poly(I:C) or 5’ppp-dsRNA. Age-matched animals administered only the adjuvants were used as controls. Two weeks later, all animals were boosted and protection was tested 2 weeks after booster immunization by the microinjection of 5,000 *Pf*CSP/*Py* sporozoites in the skin. Prior to challenge, we confirmed that the sera of mice immunized with the recombinant *Pf*CSP recognizes the *Pf*CSP/*Py* sporozoites (**Supplementary Figure 8C**).

**Figure 6:**
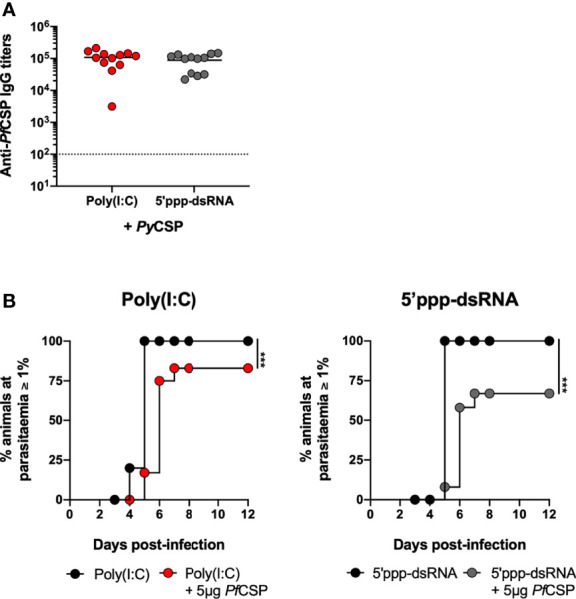
Short-term protection conferred by immunization with *Pf*CSP in combination with RLRs adjuvants. **(A, B)** Short-term protection to a skin challenge in control mice (n = 12) and mice immunized with *Pf*CSP in combination with poly(I:C) (n = 12, three independent experiments) or 5’ppp-dsRNA (n = 12, three independent experiments), 2 weeks post booster immunization. **(A)** Total anti*-Pf*CSP IgG titers in the sera of immunized mice. Symbols represent individual values and black lines the mean of each group. Dotted line represents the minimal detectable titer. **(B)** Incidence of blood stage infection after a skin challenge with 5,000 *Pf*CSP/*Py* sporozoites in control and immunized mice. Statistical significance was determined with unpaired two-tailed *t*-test **(A)**. Survival curves were plotted using Kaplan-Meier plot and statistical significance was determined using the Log-Rank (Mantel-Cox) test **(B)**. ***p ≤ 0.001.

The anti-*Pf*CSP circulating IgG titers were elevated 2 weeks after booster immunization and not different between adjuvants (**Figure 6A**). Following the challenge, 12.5% and 37.5% of sterile protection were determined for the immunizations using poly(I:C) and 5’ppp-dsRNA, respectively. Importantly, mice that become infected consistently displayed an increased prepatent period, as a minimum of 1 day, when compared with their respective adjuvant controls (**Figure 6B**). Thus, with these experiments, we have shown that immunization with *Pf*CSP and poly(I:C) or 5’ppp-dsRNA can be immunogenic and protective in mice against the *Pf*CSP-expressing *P. yoelii* parasite. Importantly, the inclusion of 5’ppp-dsRNA in the immunization with the protein led to higher overall protection than the equivalent use of poly(I:C), despite similar levels of immunogenicity.

### Immunization With 5’ppp-dsRNA Induces Less Systemic Inflammatory Mediators Than Poly(I:C)

**Figure 7:**
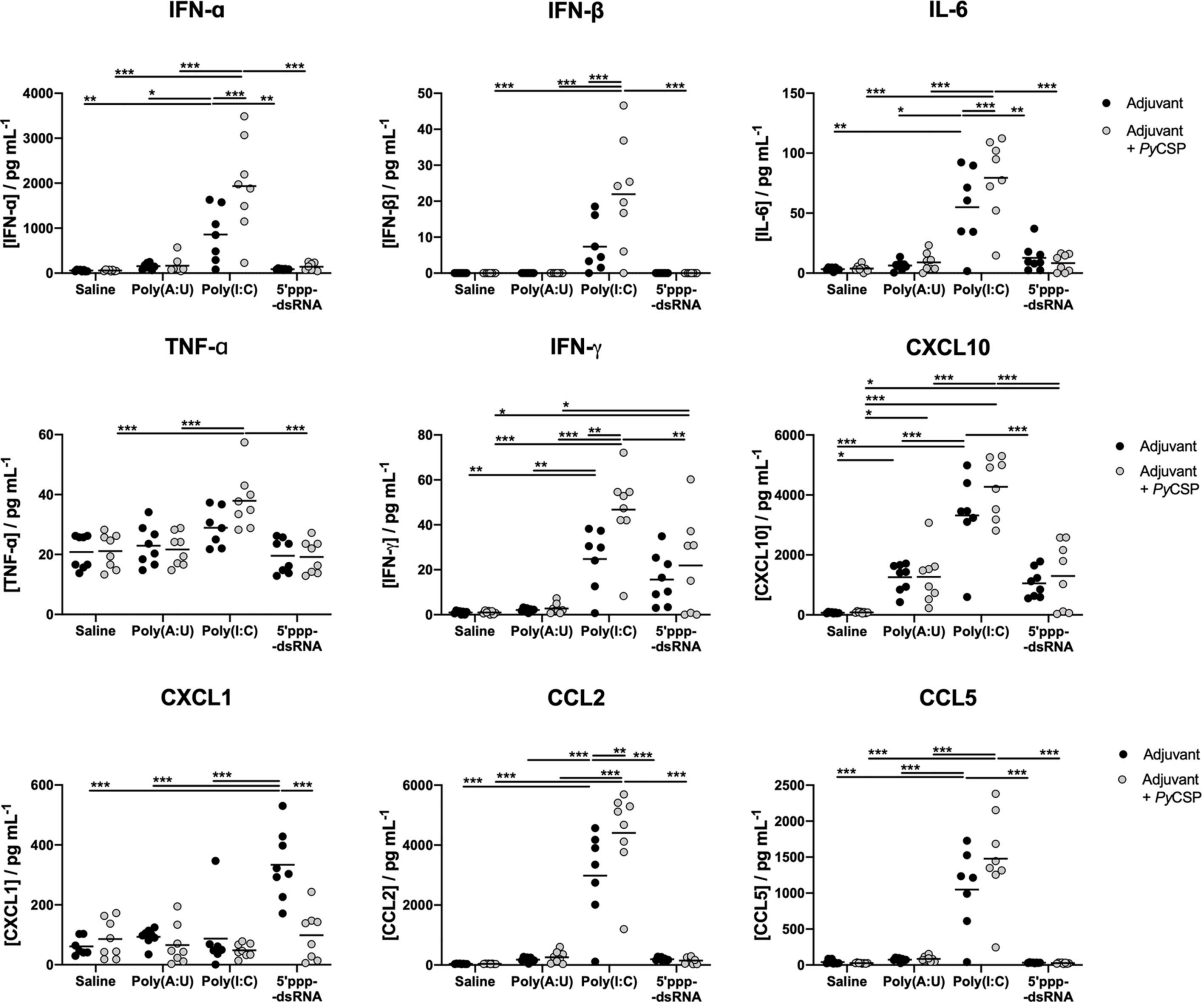
Systemic cytokine and chemokine production following administration of the different combinations of adjuvants and *Py*CSP. The concentration of several cytokines and chemokines was determined in the peripheral blood of immunized mice 5 hours after the first administration of the immunization mixtures and respective controls. Concentrations of IFN-α, IFN-β, IFN-γ, TNF-α and IL-6 and chemokines CCL2, CCL5, CXCL1 and CXCL10 were determined, using a Multiplex assay, in pg/mL. Symbols represent individual values and black lines the mean of each group for two independent experiments (n = 8). Statistical significance was determined by two-way ANOVA with Bonferroni’s multiple comparisons test. *p ≤ 0.05; **p ≤ 0.01; ***p ≤ 0.001.

To characterize the *in vivo* inflammatory profile elicited by the different adjuvants used, sera from immunized and adjuvant control mice were collected 5 hours post prime immunization and the concentration of several circulating cytokines and chemokines was quantified. The *Py*CSP administered in saline did not elicit a detectable systemic response. In general, the administration of poly(A:U) and 5’ppp-dsRNA with or without *Py*CSP had little effect for the mediators studied, except for the chemokine CXCL1, whose concentration was, on average, significantly higher for animals administered with 5’ppp-dsRNA alone. However, administration of poly(I:C), particularly when combined with *Py*CSP, resulted in significantly higher levels of all the cytokines and chemokines studied (IFN-α, IFN-β, IFN-γ, TNF-α, IL-6, CCL2, CCL5, CXCL10) when compared with all other conditions, except for CXCL1 (**Figure 7**). Similar results were observed at a later time point (10 hours) and in both time points measured after the booster (data not shown).

Our results indicate that the administration of poly(I:C) may lead to systemic inflammation, in as much as the pro-inflammatory cytokines and chemokines measured here can indicate. On the other hand, 5’ppp-dsRNA, whose use can induce equal or better protection, is, comparably, inducing a lower production of inflammatory mediators, for the time point studied. The ability of 5’ppp-dsRNA to engage the immune system to generate protection while avoiding generalized inflammation is highly desirable in an adjuvant as substances that induce systemic inflammation can raise questions about side effects.

## Discussion

The development of a highly effective malaria vaccine remains a critical priority and a goal that, despite decades of research, has yet to be achieved. Regarding subunit vaccines targeting CSP, it has become increasingly clear that antibodies can be protective, but it remains unclear how a robust long-lived, and effective response can be generated. Therefore, the development of new immunization strategies against CSP capable of promoting the generation of potent infection blocking antibodies should contribute to the improvement of vaccination against *Plasmodium*.

In the present work, we explored potential adjuvants for a vaccine against *Plasmodium*. Working within the framework of an established preclinical model of susceptibility to *Plasmodium*, as is the infection of BALB/c mice by *P. yoelii* sporozoites (46, 47), and using the major vaccine lead candidate, CSP, we tested two new adjuvants for immunization against malaria, the TLR3 agonist poly(A:U), and 5’ppp-dsRNA, a RIG-I agonist. As a benchmark, poly(I:C), a well-characterized adjuvant in the context of malaria and other vaccines (22, 28, 48, 49), was used as an activator to both receptors' pathways (50, 51).

We report that using full-length *Py*CSP adjuvanted with 5’ppp-dsRNA generated high rates of sterile protection against a stringent *P. yoelii* skin challenge, both at short- and long-term, and against mosquito bite. Protection is mediated by a strong and functional humoral response directed against sporozoites.

*Py*CSP immunization in combination with RLRs agonists was highly protective, while the protocols using poly(A:U) as an adjuvant to CSP vaccination induced remarkably lower percentages of short-term sterile protection. Interestingly, poly(A:U) has previously been described as a potent adjuvant for vaccination along with poly(I:C), in OVA immunization (32) but, in cancer therapy, poly(A:U) has been reported as being less potent than poly(I:C) when acting upon innate immune cells (31, 52). In this particular context at least, poly(A:U) was revealed to be inferior to the other adjuvants tested, including to poly(I:C). On the other hand, 5’ppp-dsRNA was revealed to be a promising adjuvant. Our data shows that the high rates of sterile protection were obtained by targeting the sporozoite in the skin, since bypassing this organ and directly inoculating sporozoites in the blood stream leads to dramatically reduced protection when compared to skin challenges. The skin has more recently gained prominence as the main site of antibody-mediated immobilization and killing of sporozoites (22, 53, 54). Accordingly, we also demonstrate that protection induced by immunization with *Py*CSP and 5’ppp-dsRNA is antibody mediated, given that passive immunization with hyperimmune sera, while unable to confer sterile protection, nonetheless conferred a significantly high degree of protection. The importance of the humoral response was underscored by results showing that T cell depletion (CD4 and CD8) immediately prior to infectious challenge was not detrimental to protection.

All adjuvanted formulations in this study induce high levels of specific IgG, but important differences in the protection that is achieved reinforce the concept that not all antibodies are equally effective in preventing infection and that the adjuvants play an important role. We found that immunization using the RLRs agonists as adjuvants, especially 5’ppp-dsRNA, induced a qualitatively superior response compared to poly(A:U). This is consistent with previous reports on the effects of the use of RLRs agonists in immunization, which have been shown to induce an enhanced germinal center reaction and affinity maturation response, resulting in a stronger humoral immunity (33, 55, 56). Although our data do not directly show an enhanced germinal center reaction in response to the protective immunization protocols, the antibodies generated displayed increased functionality *in vitro* and increased specificity for the central repeats of the protein. Another sign of an enhanced GC reaction is the accumulation of ASCs in the bone marrow, or LLPCs, over time. These cells are known to be derived from B cells that have gone through the GC reaction and affinity maturation processes before migrating to the bone marrow (44, 57). They are crucial for long-lasting protection against *Plasmodium* sporozoites as they continuously secrete antibodies to the circulation, ready to neutralize sporozoites upon infection (19). In our model, mice immunized with the protocols using the RLRs agonists accumulate more LLPCs and these correlate with the long-term titers of specific IgG, suggesting that these cells are indeed maintaining the long-term production of anti-CSP antibodies. Similar results have been reported in other immunization contexts (33). Other vaccine studies using RIG-I agonists as adjuvants also reported increased protection against different pathogens, underscored by activation of innate immunity and increase in specific (neutralizing) antibody titers (58–60), which is also reported for OVA immunization (61). However, in these studies, the RIG-I agonists are part of a formulation containing other adjuvant components or are dual agonists of other pattern recognition receptors which makes it difficult to untangle the specific effect of RIG-I/MDA5 activation.

Most importantly, mice immunized with formulations using poly(I:C) or 5’ppp-dsRNA adjuvanted CSP, are protected not only at short term but also at 12 weeks after booster immunization. However, mice immunized with *Py*CSP and 5’ppp-dsRNA, but not with poly(I:C), continue to be sterilely protected when re-challenged with sporozoites 10 weeks after the first challenge. Although in these conditions the titers of specific IgG in both groups of mice were not different, they were, nonetheless, lower when compared with the specific titers measured, in the same mice, at 2 weeks post booster immunization, just before the first challenge.

The decreased titers measured at this time point are surprising, considering that, in immunized mice which are unchallenged 12 weeks post booster immunization, the anti-CSP IgG titers are maintained over time, such that they are not different from the titers measured at an earlier time point. It has been described before that exposure to *Plasmodium* blood stage infection may lead to altered recall responses to secondary infections (62) and even lead to the deletion of both *Plasmodium*-specific and bystander LLPCs (63). Yet, in the present model, the re-infected animals were sterilely protected to the initial challenge, hence, they did not develop a detectable blood infection. Our data thus suggests that even an unproductive sporozoite infection may also impact established immunity. In light of this observation it might be important to consider that the decrease in specific antibody titers observed in clinical trials in endemic regions of other CSP-based subunit vaccines (5, 13, 17) may be due to this effect. It will be paramount to understand the mechanisms which are underlying this phenomenon as it can have wide-ranging effects for the application of these vaccines.

For other infection models, similar results have been reported. For instance, infection by *Salmonella*, through TNF-α mediated inflammation, can cause the decrease of circulation IgG titers to a previously immunizing antigen, by mobilizing LLPCs from the bone marrow without replenishment of the lost population (64). It is possible that similar effects happening in other infection models may also play a role in sporozoite infection and, indeed, the lowering of specific titers following the challenge suggests issues in the established LLPC population in the bone marrow. Additionally, although not entirely understood, it is known that the bone marrow niche of LLPCs maintains a certain plasticity, to maintain relevant immunity in the face of limited physical space (44). It has been observed that, after booster vaccination with tetanus toxoid, antigen-specific plasma cells can be observed in the blood, accompanied by an increase in the frequency of non-related ASCs and these results have been taken as evidence that competition in the bone marrow niche between the newly formed ASCs and previously established ones, led to the displacement of old cells (65). Similarly, a sporozoite infection might lead to the generation of new ASCs which can cause the displacement of the vaccine-induced *Py*CSP-specific ASCs. Thus, the established anti-CSP immunity may be replaced either by a less functional, but still CSP-specific response or, alternatively, be replaced by antibodies targeting other sporozoite antigens, which are less effective at conferring protection. Further study is necessary to pinpoint the cause of this decrease in specific titers and to understand if it might extend to other established immunity.

In addition to the high protection rates obtained in the preclinical rodent malaria model, we also report here the protection achieved in mice targeting the CSP of *P. falciparum* and using a transgenic *P. yoelii* sporozoite expressing *Pf*CSP (35). Such transgenic parasites are valuable resources for the preclinical testing of new vaccines, although it is important to stress that it remains to be elucidated how much predictive power they hold for human applications. Our results are encouraging as, even though we were unable to reconstitute the sterile protection rates observed for the wild-type model, the level of protection achieved was still significantly elevated. Human protective anti-*Pf*CSP antibodies are known to be mainly formed from specific germline alleles (42, 66, 67) and the binding of these progenitors to the protein has been found to be dependent on the existence of tryptophan at position 52, a condition that appears to be absent in the mouse counterparts to these human alleles (42).

Early clinical trials testing the systemic administration of poly(I:C) to humans described high serum interferon levels and some toxic effects, some of which were correlated with the serum level of interferon, such as hypotension and arthralgia-myalgia, but others, namely fever and nausea, were not (68, 69). Here, we report that intraperitoneal administration of poly(I:C) in mice also induces a systemic pro-inflammatory profile, limited not only to type-I interferons and interferon-gamma but also to other mediators, such as TNF-α and IL-6. Given its possible toxicity, and notwithstanding the continuation of clinical trials testing the anti-tumor effects of poly(I:C), mainly through intramuscular or intratumoral administration, less toxic alternatives to poly(I:C) have been sought, mostly related to the alteration of its formulation (49). However, we propose here that 5’ppp-dsRNA may be an equally good alternative, having displayed similar, and, under some conditions, even better, adjuvant effects without inducing as high a systemic pro-inflammatory profile. Other RIG-I agonists have also been shown to be less inflammatory and induce lower serum cytokine release, even upon intravenous administration, especially compared with poly(I:C) (60, 61).

Currently, RIG-I agonists have mostly been explored as immunotherapeutic agents against cancer (70). Results from some of these clinical trials (NCT03739138 and NCT03065023) and others (71), show that RIG-I agonists can be safely administered to humans and can be produced under Good Manufacturing Practices (GMP), demonstrating the potential for these substances to be licensed for human use. Although the necessity for a transfection reagent could be seen as a disadvantage to the use of these agonists, the aforementioned clinical trials do include some form of non-viral vector or transfection reagent to deliver the agonist studied, and thereby demonstrate these reagents can be produced under GMP and their use in humans may be possible. Another potential concern for the use of these agonists as vaccines adjuvants may be related to Single Nucleotide Polymorphisms (SNPs) of the receptor gene itself, which might make individuals under- or over-reactive to the agonist and, consequently, induce secondary effects (72). However, characterization of these SNPs and their frequency in the general population requires further study. Overall, while promising for use in human vaccines, 5’ppp-dsRNA and other RLRs agonists still require additional investigation to ensure their safety and effectiveness.

In conclusion, this is the first description of the use of 5’ppp-dsRNA as an adjuvant to a malaria vaccine. Its use as an adjuvant to CSP immunization induces strong and long-lasting sterile protection, dependent on functional antibodies and which can also withstand re-infection. This represents a step forward in the development of a vaccine against *Plasmodium*, not only for the high levels of protection achieved in a highly susceptible preclinical model but also because of the characterization of the mechanisms of action for protection may prove to be useful in other applications of 5’ppp-dsRNA.

## Data Availability Statement

The original contributions presented in the study are included in the article/**Supplementary Material**. Further inquiries can be directed to the corresponding author.

## Ethics Statement

The animal study was reviewed and approved by i3S Animal Welfare and Ethics Review Body (ORBEA) and the Portuguese national authority (DGAV), respectively.

## Author Contributions

ART, BPC and JT conceived and designed the experiments. ART, BPC, DC, MS, JR and JT performed the experiments. SG and HSD reared the mosquitoes. IK, SI, MY and MT contributed with the *Pf*CSP/*Py* line. SB contributed with plasmids and optimized conditions for recombinant protein expression. ART, BPC, RA, ACS and JT analysed the data. ART, BPC and JT drafted the manuscript. All authors reviewed the manuscript.

## Funding

The work was funded by Fundação para a Ciência e Tecnologia (FCT)/Ministério da Educação e Ciência (MEC) and FEDER through the project PTDC/SAU-PAR/31340/2017 (to JT) and the research Unit No. 4293. The authors acknowledge funding from French Government’s Investissement d’Avenir program, Laboratoire d’Excellence “Integrative Biology of Emerging Infectious Diseases” (grant no. ANR-10-LABX-62-IBEID to RA). JT, DC, MS, and AR received individual funding from FCT (CEECIND/02362/2017, SFRH/BD/123734/2016, SFRH/BD/133485/2017 and SFRH/BD/133276/2017, respectively).

## Conflict of Interest

The authors declare that the research was conducted in the absence of any commercial or financial relationships that could be construed as a potential conflict of interest.

The handling editor declared a shared affiliation with one of the authors, SB, at time of review.

## Publisher’s Note

All claims expressed in this article are solely those of the authors and do not necessarily represent those of their affiliated organizations, or those of the publisher, the editors and the reviewers. Any product that may be evaluated in this article, or claim that may be made by its manufacturer, is not guaranteed or endorsed by the publisher.
