# Supplementary material for: Immunization with CSP and a RIG-I Agonist is Effective in Inducing a Functional and Protective Humoral Response Against Plasmodium
Source: Front Immunol. 2022 May 20;13:868305. doi: 10.3389/fimmu.2022.868305 (PMC9163323; doi:10.3389/fimmu.2022.868305)
Supplement: Supplementary file 1 [file DataSheet_1.docx]

Supplementary Material

# Supplementary Figures and Tables


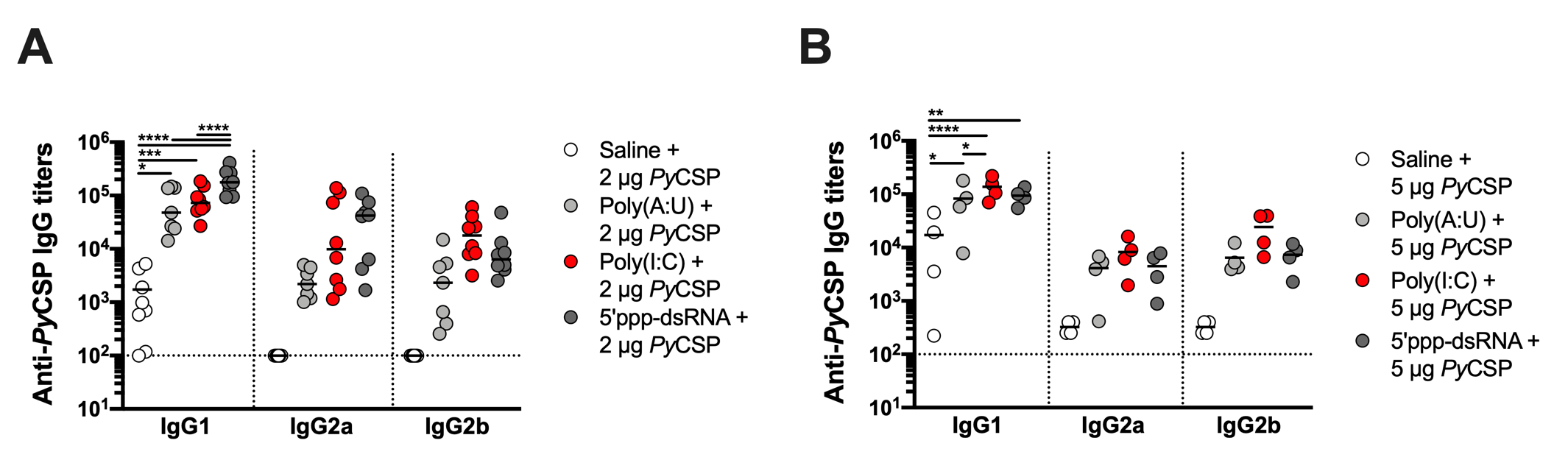


**Supplementary Figure 1.** **Short-term humoral response following immunization with *Py*CSP in combination with different adjuvants.** Anti*-Py*CSP IgG1, IgG2a, and IgG2b titers 2 weeks post booster immunization in animals immunized with **(A)** 2 μg (n=8, two independent experiments) or **(B)** 5 µg (n=4, one experiment) of *Py*CSP in saline and in combination with different adjuvants. Symbols represent individual values and black lines the mean of each group. Dotted line represents the minimal detectable titers. Statistical significance was determined using two -way ANOVA with Tukey’s multiple comparisons test. *p≤0.05; **p≤0.01; ***p≤0.001; ****p≤0.0001.

**
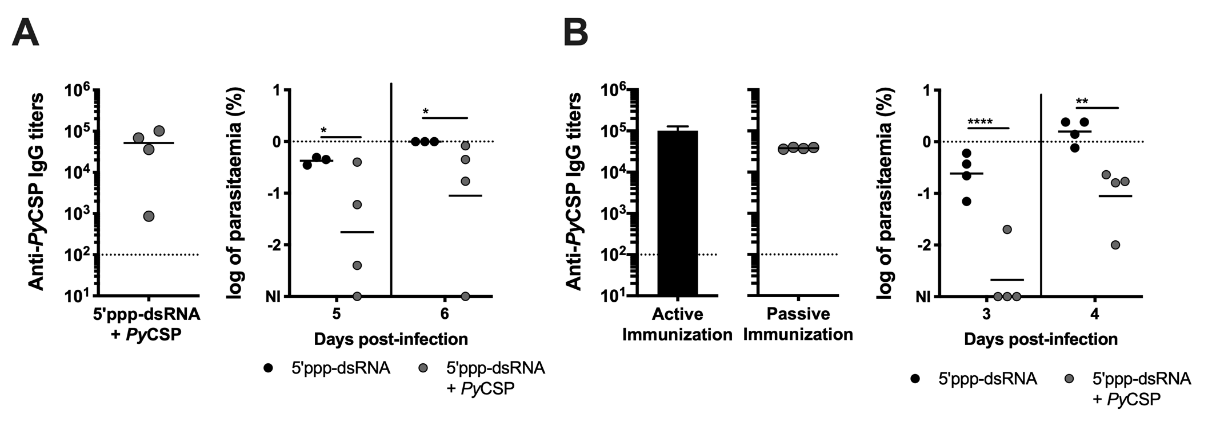
**

**Supplementary Figure 2.** **(A)** Protection to intravenous challenge in control mice (n=4) and mice immunized with *Py*CSP in combination with 5’ppp-dsRNA (n=4, one experiment), 2 weeks post booster immunization. *Left:* Anti-*Py*CSP antibody titers in the sera of immunized mice. Symbols represent individual values and black lines the mean of each group. Dotted line represents the minimal detectable titers. *Right:* Log of parasitemia in immunized and adjuvant-only control mice on days 5 and 6 post challenge with 250 sporozoites. Symbols represent individual values and black lines the mean of each group. The dotted line marks parasitemia of 1%. **(B)** Protection following passive immunization in mice which received the sera of control animals (n=4), or sera of mice immunized with *Py*CSP plus 5’ppp-dsRNA (n=4, one experiment). *Left:* Anti-*Py*CSP antibody titers in the pool of sera transferred to naïve mice (bar, “Active Immunization”) and in the sera of immunized mice 24h after transfer (symbols, “Passive Immunization”). The bar represents the mean ± SD obtained from two independent determinations. Symbols represent individual values and the black line the mean for the group. Dotted line represents the minimal detectable titers. *Right:* Log of parasitemia in control mice and passively immunized mice, at days 3 and 4 after a skin challenge with 5,000 *P. yoelii* sporozoites. Symbols represent individual values and black lines the mean of each group. The dotted line marks parasitemia of 1%. Statistical significance was determined using 2-way ANOVA with Tukey’s multiple comparisons test. *p≤0.05; **p≤0.01;****p≤0.0001.


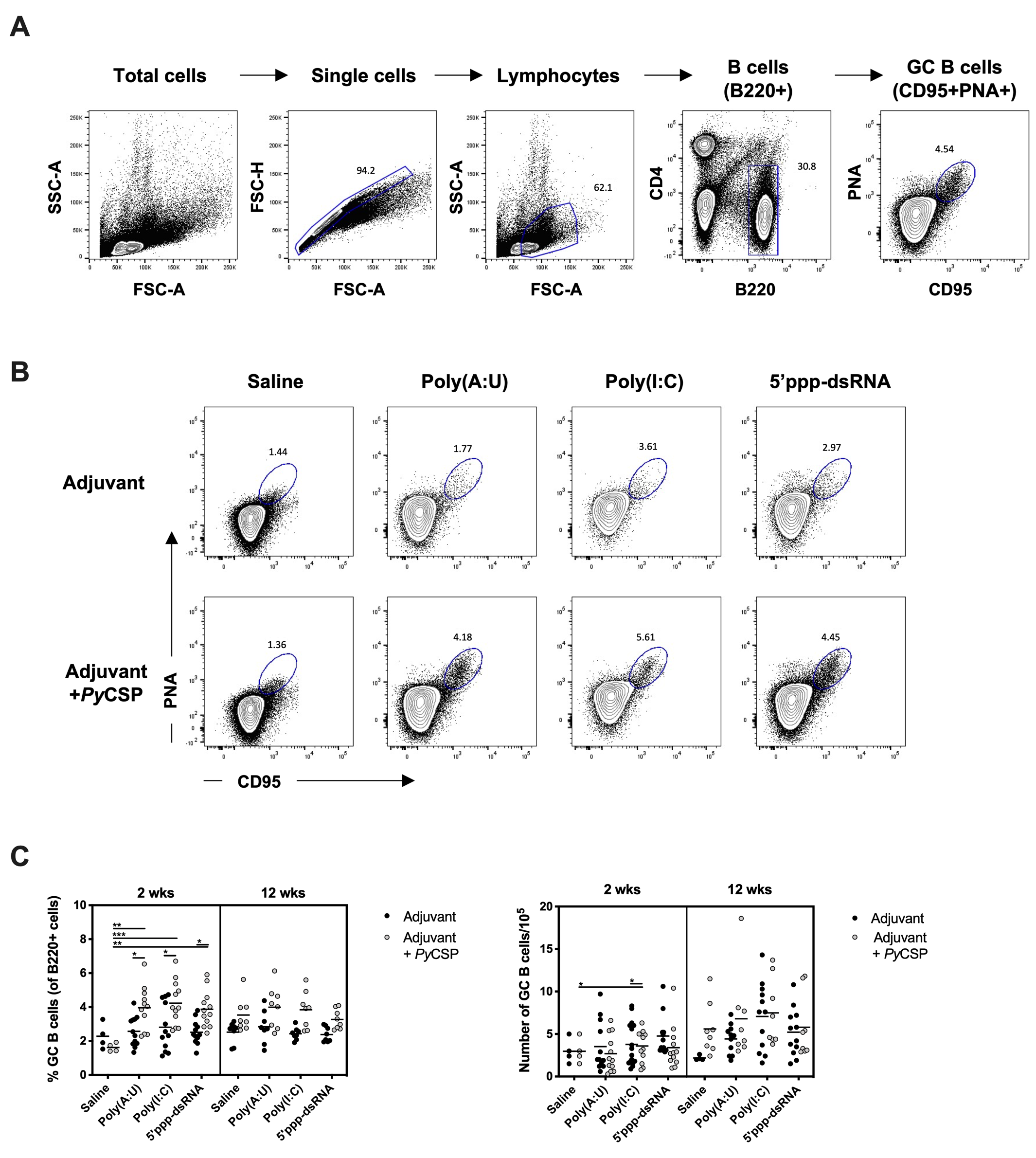


**Supplementary Figure 3.** Germinal center B cells following immunization with *Py*CSP in combination with different adjuvants. Quantification of germinal center (GC) B cells in the spleens of mice 2 (n=12, three independent experiments) and 12 (n=8, two independent experiments) weeks post booster immunization with *Py*CSP in saline and in combination with different adjuvants. **(A)** Gating strategy for flow cytometry analysis of GC B cells (B220^+^PNA^+^CD95^+^). **(B)** Representative plots for each condition. (**C)** Quantification of GC B cells in percentage (left panel) and number (right panel). Symbols represent individual values and black lines the mean of each group. Statistical significance was assessed using two-way ANOVA with Bonferroni’s multiple comparisons test. *p≤0.05, **p≤0.01; ***p≤0.001.


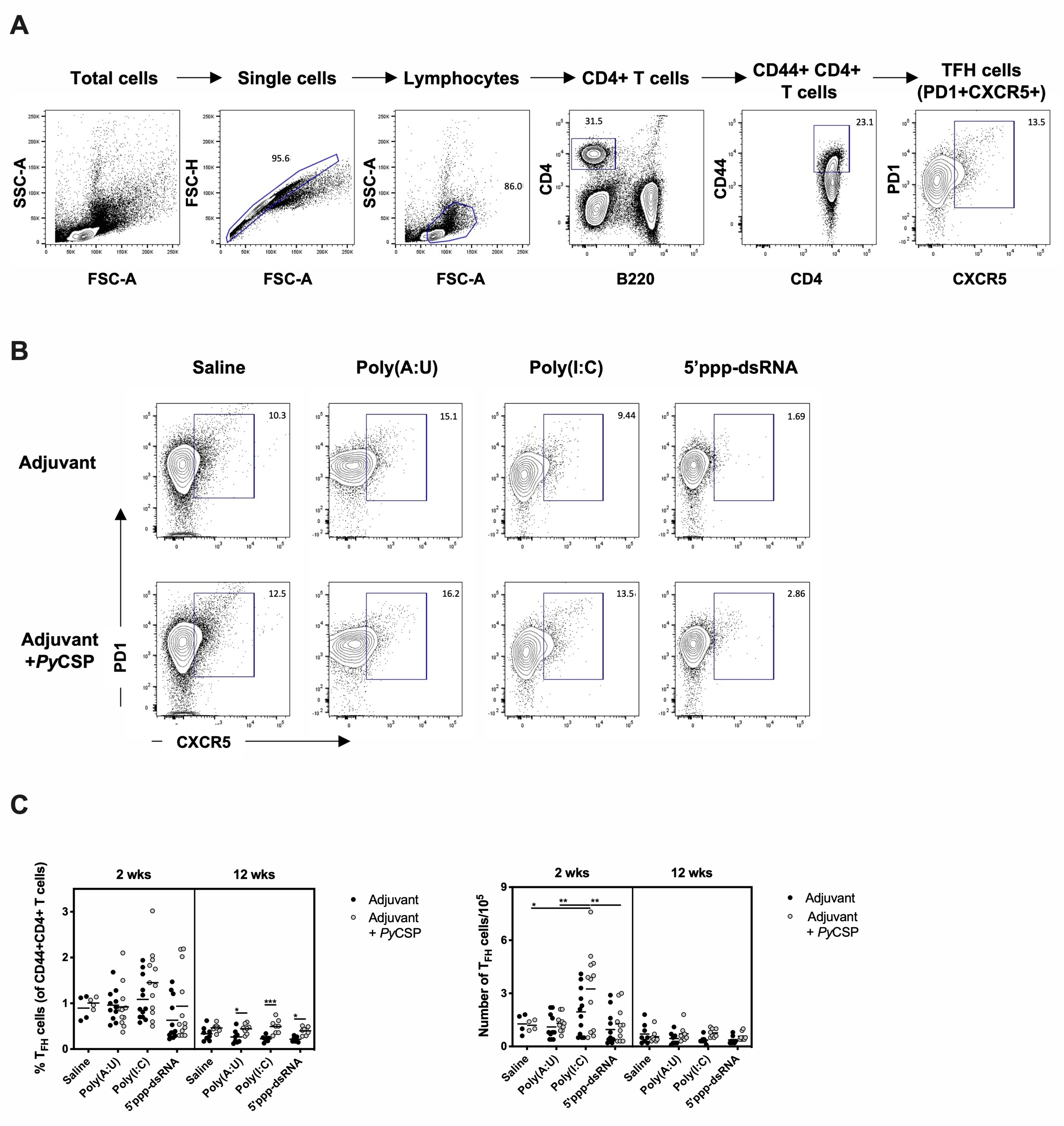


**Supplementary Figure 4. T follicular helper cells following immunization with *Py*CSP in combination with different adjuvants.** Quantification of T follicular helper (T_FH_) cells in the spleens of mice 2 (n=12, three independent experiments) and 12 (n=8, two independent experiments) weeks post booster immunization with *Py*CSP in saline and in combination with different adjuvants. **(A)** Gating strategy for flow cytometry analysis of T_FH_ cells (CD4^+^CD44^+^CXCR5^+^PD1^+^). **(B)** Representative plots for each condition. **(C)** Quantification of T_FH_ cells in percentage (left panel) and number (right panel). Symbols represent individual values and black lines the mean of each group. Statistical significance was assessed using two-way ANOVA with Bonferroni’s multiple comparisons test. *p≤0.05, **p≤0.01.


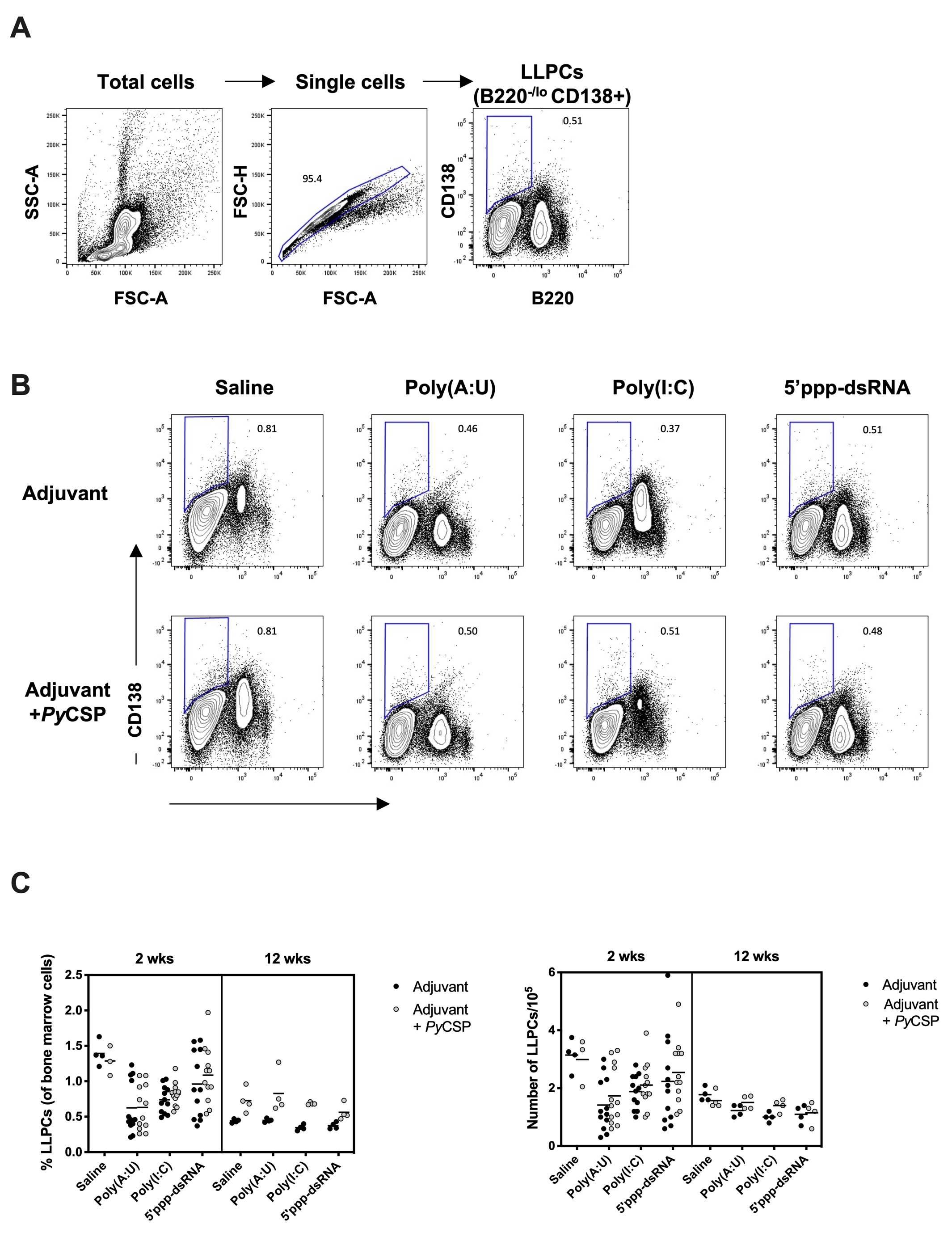


**Supplementary Figure 5. Long lived plasma cells following immunization with *Py*CSP in combination with different adjuvants.** Quantification of long-lived plasma cells (LLPCs) in the bone marrow of mice 2 (n=12, three independent experiments) and 12 (n=8, two independent experiments) weeks post booster immunization with *Py*CSP in saline and in combination with different adjuvants. **(A)** Gating strategy for flow cytometry analysis of LLPCs (B220^-/low^CD138^+^). **(B)** Representative plots for each condition. **(C)** Quantification of LLPCs in percentage (left panel) and number (right panel). Symbols represent individual values and black lines the mean of each group. Statistical significance was assessed using two-way ANOVA with Bonferroni’s multiple comparisons test.


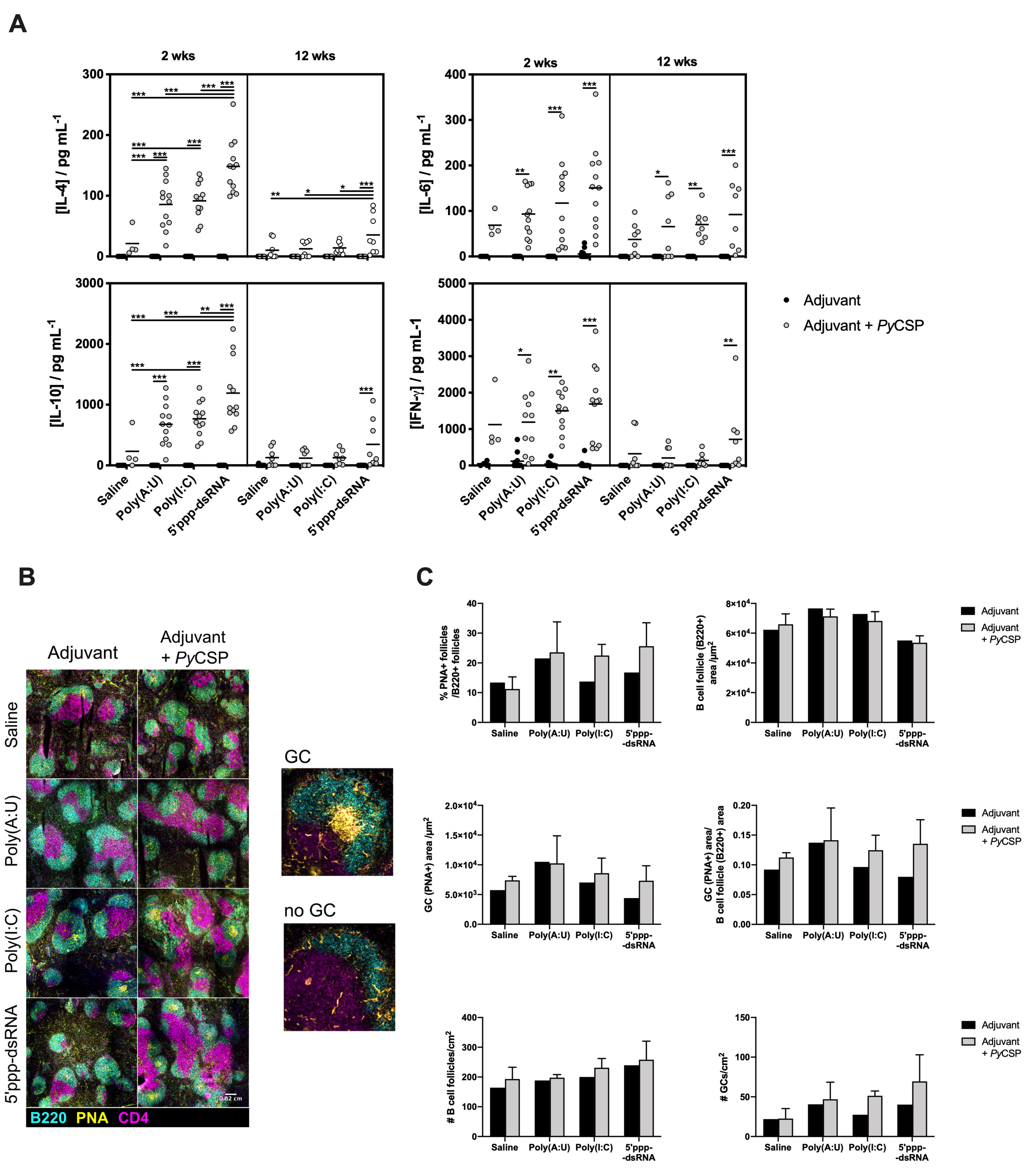


**Supplementary Figure 6. *Ex vivo* specific cytokine production and germinal center response following immunization with *Py*CSP in combination with different adjuvants.** **(A)** Quantification of *ex vivo* production of the cytokines IL-4, IL-6, IL-10, and IFN-ɣ in the supernatant of splenocytes from *Py*CSP immunized and control animals. Symbols represent individual values and black lines the mean of each group. **(B)** Representative images of spleen cryosections taken from mice immunized with *Py*CSP in saline or in combination with different adjuvants. Spleen sections were stained for B220 (cyan), PNA (yellow), and CD4 (magenta) for detection of germinal centers (GCs). GCs were defined by staining for PNA within a B cell follicle (B220+ area). **(C)** Quantification of the percentage of GC-containing B cell follicles, B cell follicles, and GC area in µm^2^, the ratio of GC/B cell follicle area, and the number of B cell follicles and GCs per cm^2^ of the spleen section. Statistical significance was determined using two-way ANOVA with Bonferroni’s multiple comparisons test. *p≤0.05; **p≤0.01; ***p≤0.001.


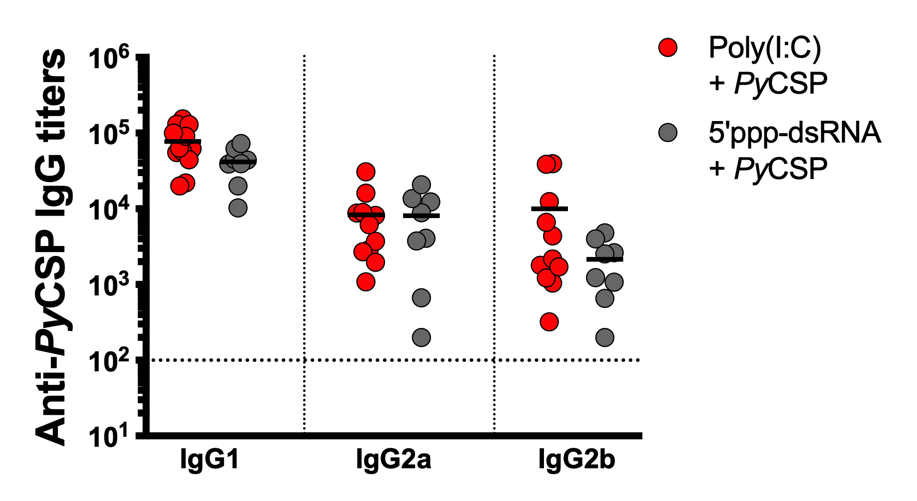


**Supplementary Figure 7. Long-term humoral response following immunization with *Py*CSP in combination with poly(I:C) and 5’ppp-dsRNA.** Anti*-Py*CSP IgG1, IgG2a, and IgG2b titers at 12 weeks post booster immunization in mice immunized with *Py*CSP in combination with poly(I:C) or 5’ppp-dsRNA. Symbols represent individual values and black lines the mean for each group. Dotted line represents the minimal detectable titer. Statistical significance was determined using two-way ANOVA with Tukey’s multiple comparisons test.


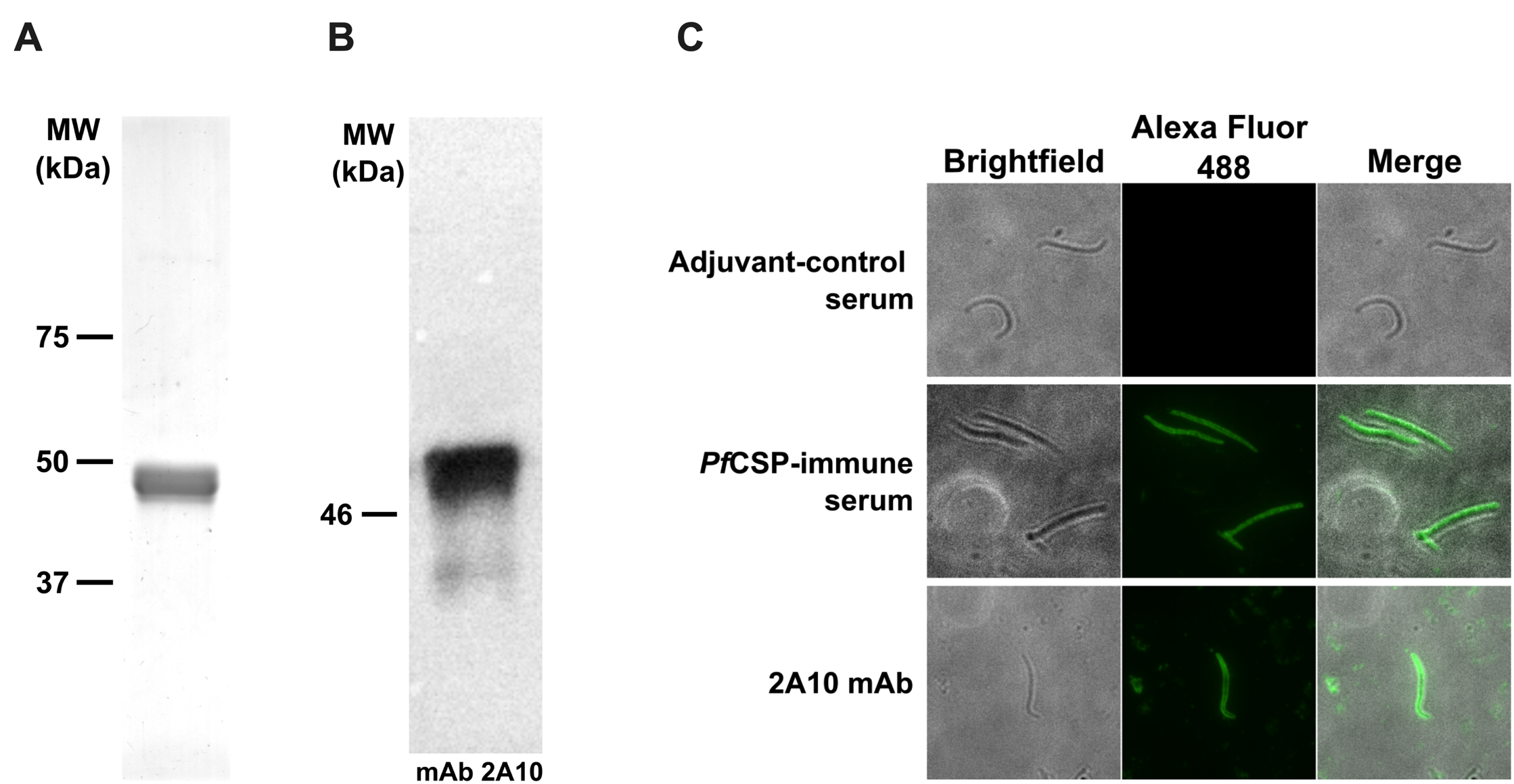


**Supplementary Figure 8. Production of recombinant *Pf*CSP. (A)** Coomassie-stained SDS-PAGE gel of purified recombinant *Pf*CSP produced in *E. coli***. (B)** Immunoblot of purified recombinant *Pf*CSP detected with the anti-*Pf*CSP repeats monoclonal antibody 2A10. **(C)** Binding of sera from control, *Pf*CSP plus 5’ppp-dsRNA immunized animals, and the anti-*Pf*CSP repeats monoclonal antibody 2A10 to *Pf*CSP/*Py* sporozoites.
